# Intermittent theta-burst stimulation improves motor function by inhibiting neuronal pyroptosis and regulating microglial polarization via TLR4/NFκB/NLRP3 signaling pathway in cerebral ischemic mice

**DOI:** 10.1186/s12974-022-02501-2

**Published:** 2022-06-11

**Authors:** Lu Luo, Meixi Liu, Yunhui Fan, Jingjun Zhang, Li Liu, Yun Li, Qiqi Zhang, Hongyu Xie, Congyu Jiang, Junfa Wu, Xiao Xiao, Yi Wu

**Affiliations:** 1 Department of Rehabilitation Medicine, Huashan Hospital, Fudan University, Shanghai, 200040 China; 2 National Center for Neurological Disorders, Shanghai, 200040 China; 3 Key Laboratory of Computational Neuroscience and Brain-Inspired Intelligence, Ministry of Education, Behavioral and Cognitive Neuroscience Center, Institute of Science and Technology for Brain-Inspired Intelligence, MOE Frontiers Center for Brain Science, Fudan University, Shanghai, 200433 China

**Keywords:** Ischemic stroke, Magnetic stimulation, Pyroptosis, Microglia, Neuroinflammation

## Abstract

**Background:**

Neuronal pyroptosis and neuroinflammation with excess microglial activation are widely involved in the early pathological process of ischemic stroke. Repetitive transcranial magnetic stimulation (rTMS), as a non-invasive neuromodulatory technique, has recently been reported to be anti-inflammatory and regulate microglial function. However, few studies have elucidated the role and mechanism of rTMS underlying regulating neuronal pyroptosis and microglial polarization.

**Methods:**

We evaluated the motor function in middle cerebral artery occlusion/reperfusion (MCAO/r) injury mice after 1-week intermittent theta-burst rTMS (iTBS) treatment in the early phase with or without depletion of microglia by colony-stimulating factor 1 receptor (CSF1R) inhibitor treatment, respectively. We further explored the morphological and molecular biological alterations associated with neuronal pyroptosis and microglial polarization via Nissl, EdU, TTC, TUNEL staining, electron microscopy, multiplex cytokine bioassays, western blot assays, immunofluorescence staining and RNA sequencing.

**Results:**

ITBS significantly protected against cerebral ischemia/reperfusion (I/R) injury-induced locomotor deficits and neuronal damage, which probably relied on the regulation of innate immune and inflammatory responses, as evidenced by RNA sequencing analysis. The peak of pyroptosis was confirmed to be later than that of apoptosis during the early phase of stroke, and pyroptosis was mainly located and more severe in the peri-infarcted area compared with apoptosis. Multiplex cytokine bioassays showed that iTBS significantly ameliorated the high levels of IL-1β, IL-17A, TNF-α, IFN-γ in MCAO/r group and elevated the level of IL-10. ITBS inhibited the expression of neuronal pyroptosis-associated proteins (i.e., Caspase1, IL-1β, IL-18, ASC, GSDMD, NLRP1) in the peri-infarcted area rather than at the border of infarcted core. KEGG enrichment analysis and further studies demonstrated that iTBS significantly shifted the microglial M1/M2 phenotype balance by curbing proinflammatory M1 activation (Iba1^+^/CD86^+^) and enhancing the anti-inflammatory M2 activation (Iba1^+^/CD206^+^) in peri-infarcted area via inhibiting TLR4/NFκB/NLRP3 signaling pathway. Depletion of microglia using CSF1R inhibitor (PLX3397) eliminated the motor functional improvements after iTBS treatment.

**Conclusions:**

rTMS could alleviate cerebral I/R injury induced locomotor deficits and neuronal pyroptosis by modulating the microglial polarization. It is expected that these data will provide novel insights into the mechanisms of rTMS protecting against cerebral I/R injury and potential targets underlying neuronal pyroptosis in the early phase of stroke.

**Supplementary Information:**

The online version contains supplementary material available at 10.1186/s12974-022-02501-2.

## Highlights

- rTMS significantly ameliorated cerebral ischemia/reperfusion injury-induced locomotor deficits and neuronal damage in the early phase probably through the anti-inflammatory mechanism.
- The peak of pyroptosis was later than that of apoptosis during the early phase of stroke, and pyroptosis was mainly located and more severe in the peri-infarcted area compared with apoptosis.
- rTMS inhibited neuronal pyroptosis in the peri-infarcted area rather than at the border of infarcted core.
- rTMS modulated microglial polarization in the peri-infarcted area via inhibiting TLR4/NFκB/NLRP3 signaling pathway.
- Depletion of microglia eliminated the motor functional improvements after rTMS treatment.

**Supplementary Information:**

The online version contains supplementary material available at 10.1186/s12974-022-02501-2.

## Introduction

Stroke is the most common cause of permanent disability, which currently affects approximately 33 million stroke survivors worldwide. Over 70% of stroke survivors suffer from motor or other neurological dysfunctions [1]. The disruption of blood supply in ischemic stroke induces widespread neuronal death in the central core of ischemic area, whereas the penumbra is surrounding areas comprising as much as half the total lesion volume and experiencing a lower level of ischemia in which neurons are functionally depressed but still viable, at least at early timepoints [2]. Under the influence of some adverse factors triggered by the primary ischemic injury, such as glial activation, neuroinflammation, and oxidative stress [3], infarcted lesions may expand over time at the expense of penumbra which will finally develop into infarcted tissue around the peri-infarcted area. Immunity and neuroinflammation appear to play an important role in delayed neuronal death after stroke [4]. Pyroptosis, defined as a highly specific inflammatory programmed cell death, differs from necrosis or apoptosis and is characterized by rapid plasma-membrane rupture and release of proinflammatory intracellular contents as well as cytokines [5]. Recently, it has been reported that neuronal pyroptosis is widely involved in the early pathological process of central nervous system (CNS) diseases, including traumatic brain injury [6], cerebral ischemia/reperfusion (I/R) injury [7] and spinal cord injury [6]. A number of publications have reported different inflammasomes activation implicated in stroke, such as NLRP1, NLRP3, and AIM2, which contribute to activation of Caspase1 that shears the precursors of IL-1β and IL-18 into their mature forms (cl.IL-1β and cl.IL-18). Meanwhile, cleaved Caspase1 cleaves Gasdermin D (GSDMD) to form pores that directly permeabilize the plasma membrane, consequently leading to rupture of plasma membrane and release of inflammatory cellular contents, including matured IL-1β and IL-18, which inducing inflammatory responses or initiating the process of inflammatory cell death [5]. Previous study revealed that the peak of neuronal apoptosis prior to that of neuronal pyroptosis and the duration of pyroptosis was much longer than that of apoptosis in spinal cord injury, suggesting that timely intervention to inhibit neuronal pyroptosis is potentially valuable [8].

Microglia, the resident immune cells in CNS, can be activated rapidly after acute ischemic stroke and are responsible for brain damage, neuronal death and inflammatory response [9]. The patterns of microglial activation vary substantially between the infarcted core and peri-infarcted area [10]. In the core area, early recruitment of anti-inflammatory microglia (M2 phenotype) may represent an endogenous effort to clean ischemic tissue and restrict brain damage. However, the endogenous recovery after stroke is usually insufficient to significantly improve long-term neurofunctional outcomes, as the M2 phagocyte response is transient and phased out within 7 days after injury. In the meantime, pro-inflammatory microglia (M1 phenotype), which are characterized by reduced phagocytosis and increased secretion of pro-inflammatory mediators, begin to predominate in the peri-infarcted area and peak around 2 weeks following stroke [11]. Therefore, the idea to change neuronal microenvironment and further inhibit neuronal pyroptosis by shifting microglial activation state from M1 to M2 in the peri-infarcted area during an early phase of ischemic injury may serve as a promising treatment strategy for ischemic stroke.

Repetitive transcranial magnetic stimulation (rTMS) is a non-invasive neuromodulation technique which has the ability to produce changes in the electrical potential of neurons and modify cortical excitability through the intact skin and skull. It has been well-accepted that high-frequency stimulation (≥ 5 Hz) and intermittent theta-burst stimulation (iTBS) improve neuron excitatory, while low-frequency stimulation (≤ 1 Hz) and continuous theta-burst stimulation (cTBS) inhibit neuron excitatory [12]. Although rTMS is widely applied in the clinical settings, the exact cellular and molecular mechanisms underlying rTMS-based therapies remain largely undetermined. Experiments in vivo and vitro revealed that rTMS could also protect neurons against death [13], and alter cerebral blood flow [14]. Among the different mechanisms involved, inflammation is one of the possible targets of rTMS effects, although few analyses have been performed so far [15]. Some recent studies have reported that non-invasive neuromodulation technique, including transcranial direct current stimulation (tDCS) [16], transcranial focused ultrasound stimulation (tFUS) [17] and rTMS [18] could also influence non-excitable cells biology, such as microglia and astroglia. However, there is no conclusive evidence to gauge the effect of rTMS on glial function from such a small number of studies with heterogeneous results, which also failed to investigate the underlying mechanisms. Therefore, the objective of the present study was to elucidate the role of neuron–microglia interactions in pathogenesis of ischemic stroke and whether rTMS could alleviate cerebral I/R injury induced locomotor deficits and neuronal pyroptosis by modulating the microglial polarization.

## Materials and methods

### Animals

Adult male C57BL/6 J mice (20–25 g) were purchased from Shanghai Jihui Laboratory Animal Care Co., Ltd., and housed in constant room temperature (22 ± 2 °C) with 50 ± 10% humidity under a 12/12 h light/dark cycle (lights on 7:00 a.m.) with food and water ad libitum for at least 1 week before the experiments. All experiments procedures were conducted according to the Animal Care and Use Committee guidelines of Huashan Hospital of Fudan University. Every effort was made to avoid/alleviate animal pain and distress and minimize the number of animals used in the project.

### Middle cerebral artery occlusion/reperfusion (MCAO/r) surgery

**Fig. 1:**
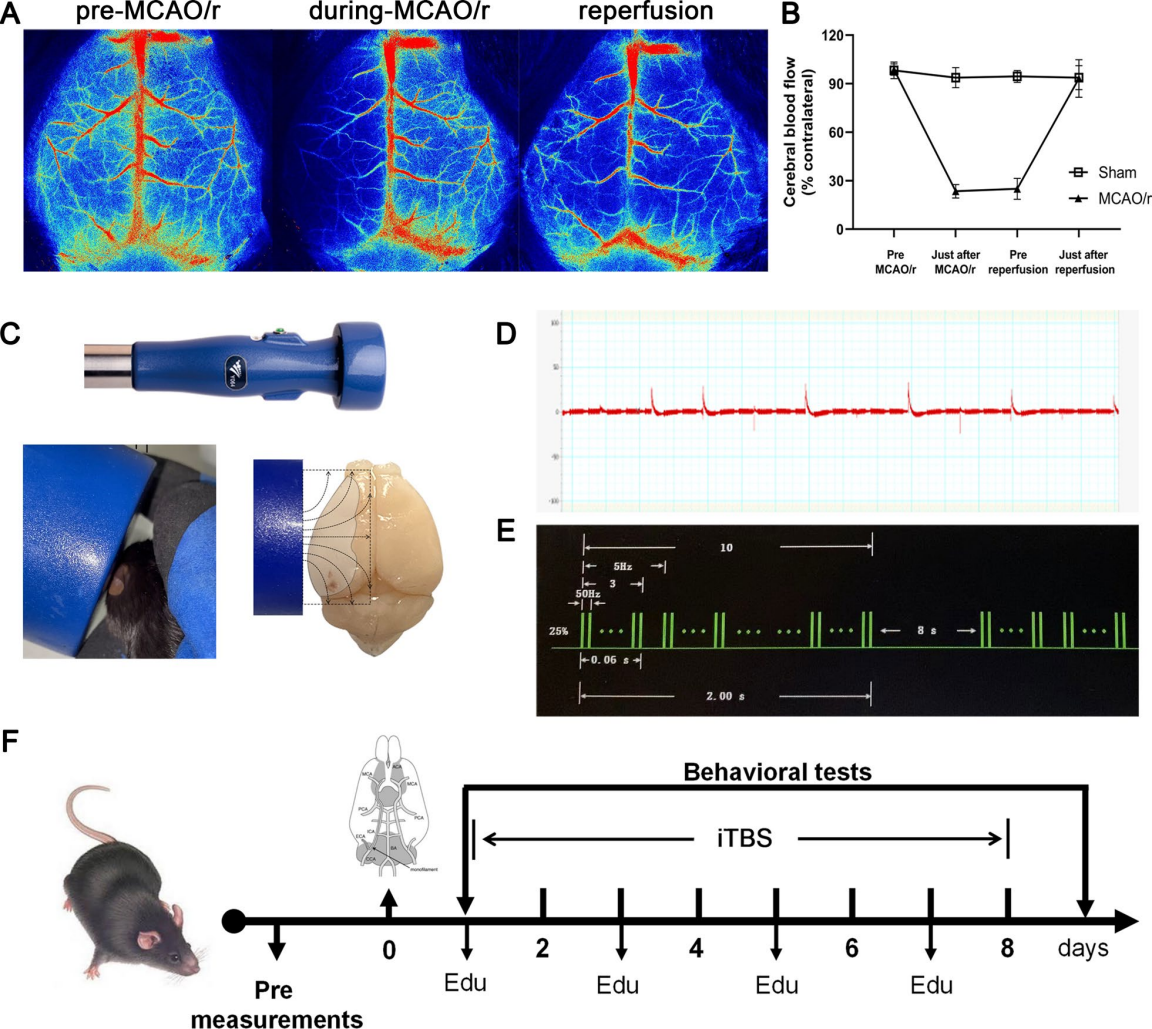
Establishment of animal model of cerebral ischemia and rTMS application. **A** MCAO/r model was established under cerebral blood flow monitored by laser speckle flowmetry. **B** Ischemic cerebral blood flow dropped below 30% of the contralateral side during surgery (*n* = 6). **C** During the stimulation, mice were un-anesthetized, gently grasped in rubber glove. The magnetic field covers most of the cortical area of the infarcted hemisphere. **D** Motor evoked potentials of the lower limb gastrocnemius muscle in mice. **E** Mice received a standard theta-burst stimulation paradigm as closely as possible to the protocol used in human studies (ten 50 Hz bursts with 3 pulses each repeated 20 times at 5 Hz intervals). **F** Experimental design of the study. Mice were subjected to MCAO/r after baseline assessments. Then, animals were randomly subdivided into Sham, MCAO/r, and iTBS groups. Following 7 continuous days of transcranial magnetic stimulation session (iTBS group) or sham stimulation (Sham and MCAO/r groups), all groups underwent post-intervention assessments. EdU was intraperitoneally administered for detecting cell proliferation every 2 days during experiments, initiated 24 h after MCAO/r surgery

MCAO/r model of left middle cerebral artery ischemia for 60 min was produced as previously described [19]. Briefly, male C57BL/6 J mice were anesthetized with 1% pentobarbital sodium (100 mg/kg. ip). The body temperature was maintained at 37.0 ± 0.3 °C during surgery via a temperature-regulated heating pad. Incisions were made at the neck midline to expose the left common carotid artery (CCA), external carotid artery (ECA) and internal carotid artery (ICA). After ECA was ligated, a silicon-coated monofilament (diameter: 0.18 ± 0.01 mm, Guangzhou Jialing Co., Ltd, China) was inserted from the left ECA, through the bifurcation of CCA, into the intracranial segment of ICA (10 ± 1 mm away from the carotid bifurcation) to block blood flow at middle cerebral artery (MCA) for 60 min. The regional cerebral blood flow (rCBF) of MCA was monitored by laser speckle flowmetry (RWD life science, Shenzhen, China; Additional file 1: Fig. S1B). A decline in ischemic rCBF ≥ 70% of the contralateral side during surgery was considered as a successful occlusion (Fig. 1A, B). Then, the monofilament was withdrawn to allow reperfusion. Sham-operated animals underwent the same anesthesia and surgical procedures except that the filament was not inserted into ICA.

### Neurobehavioral assessment

The neurological deficit level of the mice was examined using the modified neurological severity score (mNSS) 24 h after MCAO/r surgery. The full score of mNSS is 14 points, including motor (muscle state and abnormal movement), reflex and balance tests. Failure to perform one of the tests will get 1 point, and no corresponding test reflection will deduct 1 point. The overall comprehensive score is used to determine the degree of injury. MCAO/r injured mice with mNSS more than 6 points participated in the experiment.

### Repetitive transcranial magnetic stimulation application

Mice were randomly assigned to three groups: (1) Sham group (*n* = 15); (2) MCAO/r group (*n* = 25); (3) iTBS group (*n* = 25). During the stimulation, mice were un-anesthetized, gently grasped in rubber glove (Fig. 1C). To reduce stress related to handling, restraining, and stimulation, mice received a 4-day habituation to the handling and immobilization prior to starting the intervention. All administrations were performed with a magnetic stimulator (CCY-II, Wuhan Yiruide Medical Equipment, Wuhan, China; Additional file 1: Fig. S1A) connected to a round coil (diameter: 6.5 cm), positioned in most cortical areas of the infarcted hemisphere (Fig. 1C). Motor evoked potentials (MEPs) were recorded from the contralateral gastrocnemius muscle via needle electromyography electrodes. Thereafter, the stimulation intensity was increased by 5%. Resting motor threshold (RMT) was defined as the lowest stimulator output when muscle twitches or peak-to-peak amplitude of MEPs is greater than 50 μV in at least 5 out of 10 consecutive trials (Fig. 1D). In our study, the average RMT was 25% of the maximum stimulator output strength (range 20–30%). The stimulation intensity was set at 100% of the average RMT of mice. The motor threshold was randomly checked at the beginning of the stimulation session for each animal.

In formal experiment, mice in the iTBS group receive iTBS (ten 50 Hz bursts with 3 pulses each repeated 20 times at 5 Hz intervals; Fig. 1E) twice per day (9:00 a.m.–11:00 a.m., 3:00 p.m.–5:00 p.m.) beginning at 36 h after MCAO/r injury for 7 continuous days (Fig. 1F). The stimulation parameters were chosen to be as closely as possible to the rTMS protocol used in clinic setting. For the sham stimulation groups (Sham and MCAO/r groups), we used the same stimulation protocol but with the coil placed 15 cm above the animal’s head.

### Colony-stimulating factor 1 receptor (CSF1R) inhibitor treatment

To deplete microglia in vivo, mice received the CSF1R inhibitor PLX3397 (AdooQ BioScience, A15520), which were mixed into AIN-76A standard chow at 290 mg/kg. AIN-76A standard chow alone served as the control. Mice were placed on the chow diet ad libitum for 3 weeks prior to induction of MCAO/r and the diet was maintained until the mice were killed. During the PLX3397 treatment, mice had no obvious behavioral or health problems except for patchy whitening of the fur.

### Behavioral tests

The behavioral tests were performed 1 day before and 8 days after MCAO/r surgery. The test sequence was as follows: (1) Open field test; (2) Rotarod test; (3) Cylinder test; (4) Inverted wire mesh grid grip test; (5) CatWalk XT gait test. Keeping a 2-h interval between each test.

*Open field test* The experimental device consists of a plastic test box (40 × 40 × 50 cm) and an automatic video system. The mouse was placed into the center of the open field to record its free movement for 5 min. The distance moved and mean velocity were recorded and analyzed [20].

*Rotarod test* The pre-training test (to establish a performance baseline) was performed 4 times per day for 3 consecutive days. The rotating rod accelerates from 5 revolutions per minute (RPM) until it rotates at a constant speed after 40 RPM within 90 s. Each test lasts for a maximum of 5 min, and rests for 3 min between tests to avoid fatigue. Record the latency of each mouse falling from the rod in each test and calculate the average latency as the final result. If the mouse grabs the revolver for 2 laps, stop timing [21].

*Cylinder test* Mouse was placed in a transparent cylinder (10 cm in diameter and 15 cm in height), and record the use of forelimbs in the exploratory behavior within 5 min. Place a mirror at the appropriate position to ensure that the forelimb activity can be recorded even when the mouse was turned back to the camera lens. The evaluator used a video recorder with slow-motion and clear freeze-frame functions to record and score. When analyzing the behavior in the cylinder, record the number of times that the right paw, the left paw, and both paws touch the cylinder wall simultaneously. Data were analyzed using asymmetry, which was calculated as follows: (left + 0.5 × both)/(right + left + both) × 100% [21].

*Inverted wire mesh grid grip test* Mouse was gently placed on a metal wire mesh grid with 0.8 cm gaps (12 mm in square, 1 mm in diameter). After the mice stood steadily, the mesh was rotated slowly at a steady speed to an inverted position and suspended 40 cm above a cage. Record the latency of each mouse falling from the mesh. Each testing session included 3 trials with a 5-min delay interval, and calculate the average latency as the final result [22].

*CatWalk XT gait test* In this study, a 150 cm-long small animal glass track CatWalk XT (Noldus, Netherlands, Wageningen) system was used. A high-speed camera was placed under the glass plate to capture images of mouse paw changes for gait data collection. In the experiment, the mouse was placed on the runway and passed smoothly, and it had to be completed within 8 s. The maximum speed change was 60%. At least 3 movement averages that met this standard were recorded in each round. The gait analysis indicators selected in the study included standing time, limb swing time, duty cycle, average running speed, stride length, mean intensity of the complete paw. In addition, the average running speed and running time of mice were analyzed as a whole [23].

### Experimental material preparation

Mice were deeply anesthetized by 1% pentobarbital sodium (100 mg/kg. ip). For western blotting, cytokine bioassays, RNA sequencing, mice in each group were sacrificed and brain tissue of the infarcted cortex were quickly removed, placed in Eppendorf tubes, frozen in liquid nitrogen, and stored at − 80 °C for further use. For immunohistochemistry, the mice were transcardially perfused with 50 ml of phosphate buffered saline (PBS) and then fixed with 50 ml 4% paraformaldehyde (PFA) solution. The brain was removed, post-fixed for 24 h in the same fixative and cryoprotected 24 h at 4 °C in 30% sucrose solution. After then, the tissue blocks were embedded in paraffin for further use. For electron microscopy, the peri-infarcted brain tissue (0.2 × 0.2 × 0.2 cm) was fixed with 2.5% glutaraldehyde.

### Western blot assays

Brain tissue was homogenized with an electric homogenizer in RIPA lysis buffer (150 mM sodium chloride, 1.0% Triton X-100, 0.5% sodium deoxycholate, 0.1% SDS, 50 mM Tris, pH 8.0). Centrifuge for 10 min at 12,000 rpm at 4 °C in a microcentrifuge and aspirate the supernatant. Protein concentration was measured with the BCA protein assay kit (Epizyme Biotech, China). Denatured proteins (30 μg) were separated in SDS–PAGE gel (10%, Epizyme Biotech, China) by electrophoresis under constant voltage (120 V), and further transferred onto a PVDF membrane (Millipore, USA). After blocking non-specific binding sites with a 5% BSA for 1 h at room temperature (RT), the membranes were incubated overnight at 4 °C with the appropriate primary antibody diluted in blocking solution, including pre.Caspase1, cl.Caspase1, pre.IL-1β, cl.IL-1β, IL-18, GSDMD, ASC, NLRP1, Iba-1, GFAP, NeuN, CD86, CD206, iNOS, Arg1, TLR4, NFκB, p-NFκB, NLRP3 and β-actin (Additional file 1: Table). After three times washes with TBS-T buffer (10 mM Tris, 150 mM NaCl, 0.05% Tween-20, pH 7.5), the blots were incubated for 2 h at RT with secondary antibodies: a horseradish peroxidase (HRP)-conjugated goat anti-mouse or anti-rabbit IgG (1:5000, Bioss). The membranes were developed with ECL reagents (Tanon, China) and imaged via UVP gel imaging system (UVP, USA). The relative band intensity was quantitative analyzed by Image J software (NIH, USA) and then normalized to the loading control (β-actin).

### Multiplex cytokine bioassays

Cytokines and chemokines in infarcted cortex tissue were measured and quantified using the LEGENDplex™ mouse inflammation panel (BioLegend, 740446) according to the manufacturer's instructions, which allowed simultaneous quantification of 13 cytokines, including IL-1α, IL-1β, IL-6, IL-10, IL-12p70, IL-17A, IL-23, IL-27, MCP-1, IFN-β, IFN-γ, TNF-α, and GM-CSF. All data were collected on an LSRFortessa and analyzed using LEGENDplex™ software (BioLegend).

### Nissl staining

Nissl staining was performed as previously described [24]. Briefly, sections were degreased through graded alcohol (70%, 95% and 100% alcohol) for 3 min, respectively, and then hydrated through graded alcohol (95%, 70% and 50% alcohol) for 3 min, respectively. Subsequently, the sections were stained in 0.1% toluidine blue solution for 20 min, quickly rinsed in distilled water and differentiated in 95% ethyl alcohol for 15 min.

### Immunofluorescence staining

For immunofluorescence staining, the peri-infarcted brain area of coronal slice was chosen for imaging and analysis. Briefly, coronal cryotome section (30 µm) were washed with 0.5% Triton X-100 for 10 min, and blocked with 5% bovine serum albumin (BSA) (Sigma) and 0.5% Triton X-100 (Sigma) in PBS for 1 h at RT. The following primary antibodies were used: NeuN, MAP2, Caspase1, GSDMD, ASC, IL-1β, NLRP1, Iba-1, GFAP, CD86, CD206 (Additional file 1: Table). Sections were then washed three times with PBS at RT, followed by incubation with appropriate secondary antibodies conjugated to Alexa Fluor (Thermo Fisher Scientific) for 1.5 h. After washed, the sections were mounted and coverslipped in Vectashield mounting medium with DAPI (Vector Laboratories). The sections were observed with Olympus Fluorview-3000 confocal microscope (Olympus Optical, Ltd., Japan) and quantitative analyzed by Fiji software (National Institutes of Health, USA). MAP2 expression was analyzed and expressed as mean fluorescent intensity. Cell counts on the examined sections were then averaged to provide a single value for the specific group. In the counting studies of double staining labeled cells (NeuN/Caspase1, NeuN/GSDMD, NeuN/IL-1β, MAP2/GSDMD, MAP2/ASC, NLRP1/GSDMD, Iba1/CD86, and Iba1/CD206), the proportion (%) of co-labeled cells were counted in 3 representative capture views of the peri-infarcted brain areas from each animal.

### RNA sequencing and differentially expressed genes analysis

Total RNA was extracted using the TRIzol reagent (Invitrogen, Carlsbad, CA, USA) according to the manufacturer’s protocol. RNA purity and quantification were evaluated using the NanoDrop ND-1000 (NanoDrop, Wilmington, DE, USA). RNA integrity was assessed using the Agilent 2100 Bioanalyzer (Agilent Technologies, Santa Clara, CA, USA). Then the libraries were constructed using TruSeq Stranded mRNA LT Sample Prep Kit (Illumina, San Diego, CA, USA) according to the manufacturer’s instructions. The transcriptome sequencing and analysis were conducted by LC-Bio Technology CO., Ltd. (Hangzhou, China). The libraries were sequenced on an Illumina HiSeq X Ten platform and 150 bp paired-end reads were generated. Raw data (raw reads) of fastq format were first processed using Trimmomatic [25] and the low-quality reads were removed to obtain the clean reads. The clean reads were mapped to the mus_musculus (GRCm38) using HISAT2 (https://ccb.jhu.edu/software/hisat2) [26]. FPKM [27] of each gene was calculated using Cufflinks [28], and the read counts of each gene were obtained by HTSeq-count [29]. Differential expression analysis was performed using the DESeq (2012) R package [30]. *P* value < 0.05 and foldchange > 2.0 was set as the threshold for significantly differential expression. Hierarchical cluster analysis of differentially expressed genes (DEGs) was performed to demonstrate the expression pattern of genes in different groups and samples. Gene Ontology (GO) enrichment and Kyoto Encyclopedia of Genes and Genomes (KEGG) [31] pathway enrichment analysis of DEGs were performed, respectively, using R based on the hypergeometric distribution.

### Electron microscopy

Ultrastructural changes of nerve cells in the peri-infarcted brain area were assessed with transmission electron microscopy. Slices were embedded with epoxy resin, stained with 4% uranylacetate—0.3% lead citrate and observed with transmission electron microscope (HITACHI HT 7800 120kv).

### 5-Ethynyl-2′-deoxyuridine (EdU) labeling, terminal deoxynucleotidyl transferase mediated dUTP nick end labeling (TUNEL) staining and 2,3,5-triphenyltetrazolium chloride (TTC) staining

To examine cell proliferation, EdU (50 mg/kg body weight, Beyotime, China) was intraperitoneally administered to each animal every 2 days over the 8-day period, initiated 24 h after MCAO/r surgery. EdU staining was performed with the Edu-488 cell proliferation detection kit (Beyotime, China). Briefly, frozen slides were sequentially incubated with pre-configured click reaction reagent (click reaction buffer: CuSO4: azide 488: click additive solution = 430: 20: 1: 50). TUNEL staining was performed to measure apoptosis with the TUNEL apoptosis assay kit according to manufacturer protocol (Beyotime, China). Briefly, frozen slides were sequentially incubated with pre-configured TUNEL detection reagent (terminal deoxynucleotidyl transferase: FITC–dUTP = 1: 9). The apoptosis index is calculated by dividing the number of TUNEL-positive cells by the total number of cells per-field of view. The fluorescence signal was detected by laser confocal microscope (Olympus Optical, Ltd., Japan). For TTC staining, brain tissues were cut into six sections in the coronal plane (2 mm), and then dipped in 2% TTC (Solarbio, China) for 30 min at 37 °C. The infarcted volume was measured and presented as a percentage of the non-ischemic hemisphere to correct for edema.

### Statistical analysis

Statistical tests were done on SPSS 23.0 statistical software (SPSS, Chicago, IL, USA) and GraphPad Prism 9.0 (GraphPad Software Inc., San Diego, CA, USA) using one-way or two-way analysis of variance (ANOVA) for multiple comparisons followed by Tukey’s or Bonferroni’s post hoc test. Statistical significance between two groups was determined with unpaired Student’s *t* test. The survival curve was analyzed by Log-rank (Mantel–Cox) test. A probability of 0.05 or less was considered statistically significant.

## Results

### ITBS treatment promoted motor functional recovery in cerebral I/R injury mice after acute stroke

**Fig. 2:**
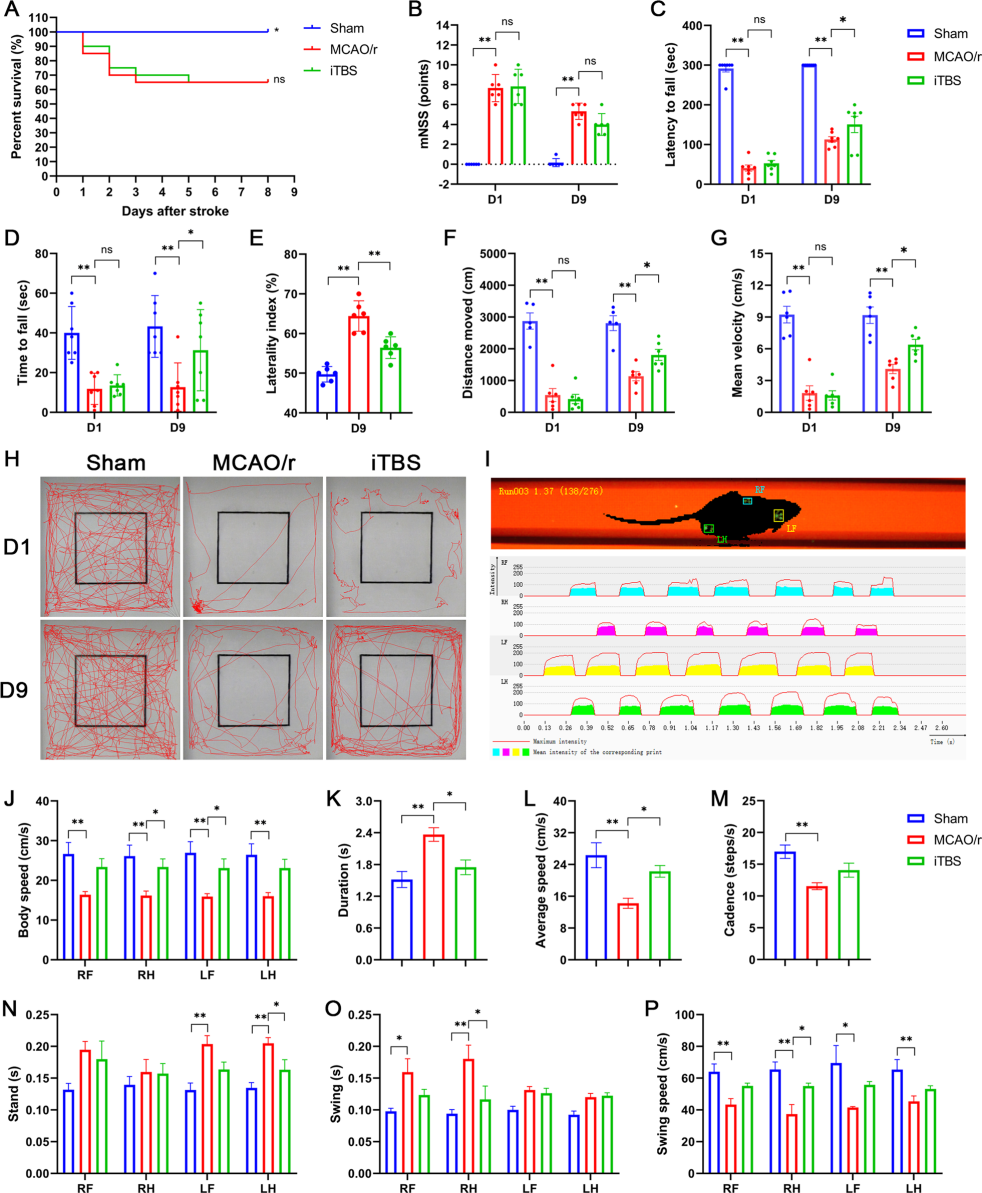
ITBS treatment promoted motor functional recovery in cerebral I/R injury mice after acute stroke. **A** Survival curve among Sham, MCAO/r and iTBS groups during 8-day observation period. No statistically significant difference in mortality data was observed between MCAO/r and iTBS groups at the end of experiment. Non-significant (ns), *P < 0.05 as determined by Log-rank (Mantel–Cox) test. **B** Cerebral ischemia induced higher neurological score, while iTBS failed to attenuate that. MCAO/r group displayed lower time to fall in rotarod test **(C)**, inverted wire mesh grid grip test **(D)**, higher laterality index in cylinder test **(E)**, and lower distance move **(F)**, mean velocity **(G)** in open filed test. ITBS significantly improved the motor behavior above. **(H)** Illustration of motion trajectory among Sham, MCAO/r, iTBS groups at 1 day (D1) and 9 days (D9) after stroke. **I** Schematic diagram of labeled footprint and footprint intensities charts. MCAO/r group displayed significant decreases in body speed **(J)**, average speed **(L)**, cadence **(M)**, swing speed **(P)** and significant increases in duration **(K)**, stand time of left limbs **(N)**, swing time of right limbs **(O)**. ITBS significantly increased body speed of right hindlimb, left forelimb **(J)**, average speed **(L)**, swing speed of right limb **(P)** and significantly reduced duration **(K)**, stand time of left hindlimb **(N)**, swing time of right hindlimb **(P)**. Values are expressed as the mean ± SEM of the mean (*n* = 6). *P < 0.05, **P < 0.01 as determined by one-way ANOVA (Tukey’s multiple comparison test) and two-way ANOVA (Bonferroni’s multiple comparison test). *RF* right forelimb, *RH* right hindlimb, *LF* left forelimb, *LH* left hindlimb

We examined the mortality of each group recorded daily for 8-day period (Fig. 2A). Mice in the Sham group showed 100% survival within the whole observation period. Three days after stroke, 35% of the cerebral I/R injury mice in the MCAO/r group had died, compared to 30% of the iTBS-treated mice, which also reached the same mortality rate as MCAO/r group at 5^th^ day post-stroke. Although no statistically significant difference (*P* = 0.94) in mortality data was observed between MCAO/r and iTBS groups at the end of experiment, iTBS treatment showed a trend of delaying the death of cerebral I/R injury mice. In the functional tests, ischemic animals committed severe motor deficit as evidenced by higher neurological score compared with sham-operated mice (*P* < 0.01). There was no statistically significant difference between MCAO/r and iTBS groups at both 1 day (D1) (*P* = 0.97) and 9 days (D9) after stroke (*P* = 0.10), which might due to a natural recovery of the animals after cerebral ischemia (Fig. 2B). In rotarod test (Fig. 2C), inverted wire mesh grid grip test (Fig. 2D) and cylinder test (Fig. 2E), a lower time to fall (*P* < 0.01) and higher laterality index (*P* < 0.01) were observed in the MCAO/r group before and after sham iTBS in relation to the Sham group, suggesting impairment of the strength and motor coordination induced by cerebral ischemia. As expected, the locomotor deficits of cerebral I/R injury mice treated with iTBS were significantly ameliorated, as evidenced by the improved time to fall (*P* < 0.05) and reduced laterality index (*P* < 0.01). These behavioral improvements were further supported by the data analysis of the increased spontaneous exploration behavior evaluated by open-field test (Fig. 2H), showing significant increase in distance of motion trajectory (*P* < 0.05; Fig. 2F) and mean velocity (*P* < 0.05; Fig. 2G) after iTBS treatment.

To evaluate the effect of iTBS treatment on gait impairment, we analyzed the run parameters after iTBS using the CatWalk automated gait analysis system (Fig. 2I). MCAO/r group displayed significant decreases (*P* < 0.01) in kinetic parameters (body speed, average speed, swing speed, cadence) and significant increases (*P* < 0.01) in temporal parameters (duration, stand time of left limbs, swing time of right limbs) compared with Sham group. There were significant increases in average speed, body speed of right hindlimb, left forelimb, and swing speed of right limb after iTBS (*P* < 0.05). Furthermore, iTBS also significantly reduced duration, stand time of left hindlimb, and swing time of right hindlimb (*P* < 0.05) (Fig. 2J–P). Taken together, these findings confirm that iTBS significantly ameliorates motor behavioral deficits and gait impairment after acute ischemic stroke.

### ITBS protected against cerebral ischemia-induced neuronal damage in the early phase probably through the anti-inflammatory mechanism

**Fig. 3:**
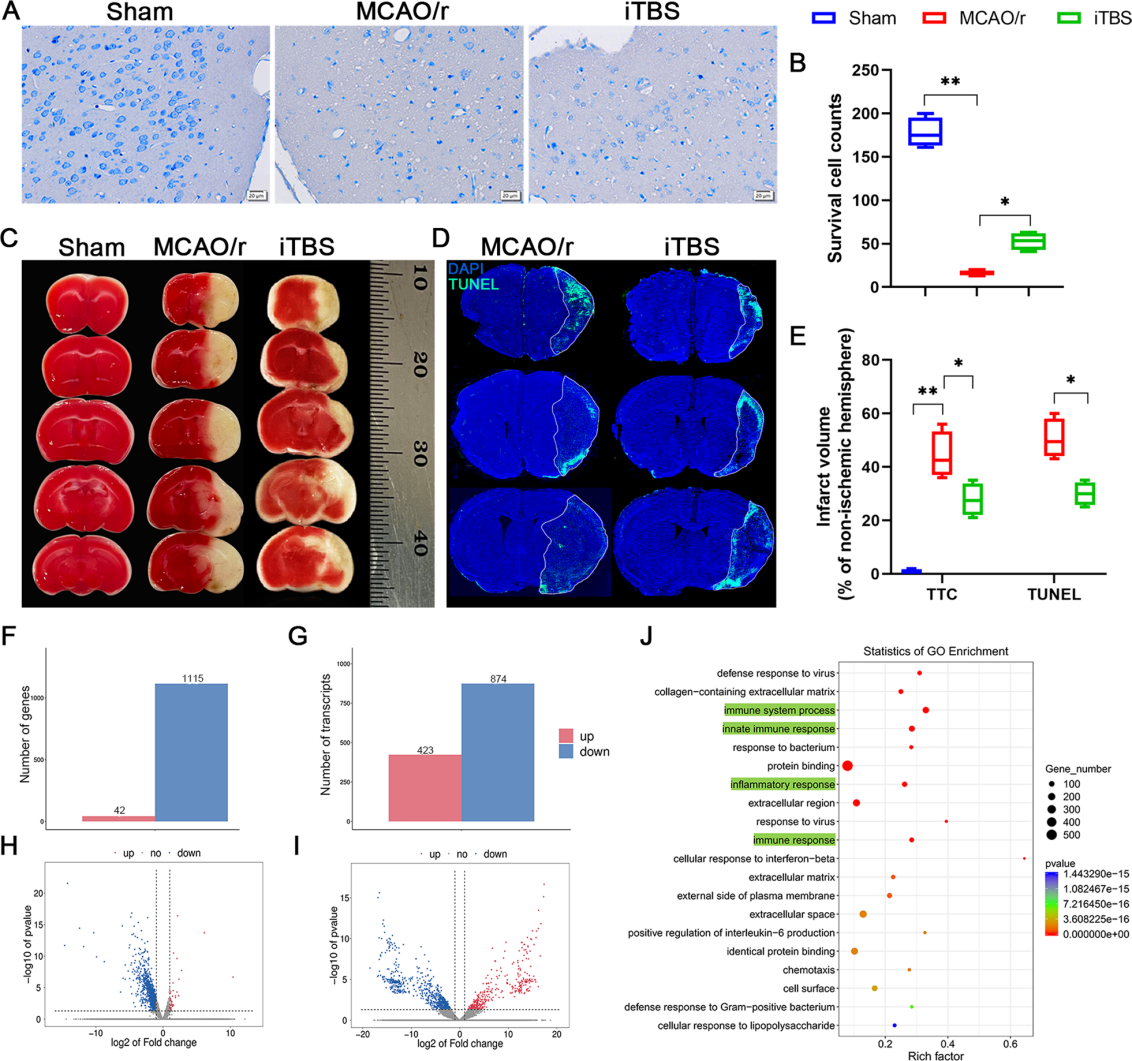
Anti-inflammatory mechanism of iTBS protecting against cerebral ischemia-induced neuronal damage. **A** Histological images of Nissl staining evaluated the neuronal survival in the ipsilateral cortex. Scale bar = 20 μm. **B** Quantitative analysis showed that iTBS significantly enhanced survival cells. **C** TTC staining and **D** TUNEL staining showed the infarcted size relative to non-ischemic hemisphere after cerebral I/R injury. **E** Quantitative analysis showed that iTBS significantly reduced infarcted volume. Values are expressed as the mean and 95% confidence interval (*n* = 4). *P < 0.05, **P < 0.01 as determined by one-way ANOVA (Tukey's multiple comparison test). RNA-seq showed 1157 differentially expressed genes with 42 genes up-regulated, 1115 genes down-regulated **(F)** and 1297 differentially transcripts with 423 transcripts up-regulated, 874 transcripts down-regulated **(G)** between MCAO/r and iTBS groups, respectively (*n* = 3). Volcano map of differentially expressed genes **(H)** and transcripts **(I)** between MCAO/r and iTBS groups. Gray represents the genes with insignificant difference. Red and blue represent the genes with significant difference. **J** GO term enrichment analysis of differentially expressed genes revealed that iTBS protected against cerebral I/R injury possibly by regulating immune system process and innate immune responses

Nissl staining showed that neuronal cells in the MCAO/r group were loosely arranged or missing, and Nissl bodies were lightly stained or even dissolved compared with that in Sham and iTBS groups, indicating neuronal injury-induced by cerebral ischemia. The number of survival cells was significantly increased in iTBS group (*P* < 0.01; Fig. 3A, B). TTC staining showed a significant increase in infarcted volume (%) relative to non-ischemic hemisphere after cerebral I/R injury in the MCAO/r group (Sham, 0.75 ± 0.48, MCAO/r, 44.25 ± 4.33; *P* < 0.01), which was significantly diminished after iTBS treatment (iTBS, 27.75 ± 3.04; *P* < 0.05; Fig. 3C, E). TUNEL staining also showed a similar reduction in infarcted volume between MCAO/r and iTBS groups (MCAO/r, 50.50 ± 3.66; iTBS, 30.00 ± 2.20; *P* < 0.05; Fig. 3D, E). EdU labeling in vivo was used to observe whether iTBS induced neurogenesis. Our results indicated a significant increase in the number of EdU^+^ cells in hippocampal dentate gyrus (DG) (*P* < 0.01; Additional file 1: Fig. S1C) and non-significant increase in subventricular zone (SVZ) (*P* = 0.27; Additional file 1: Fig. S1D) in MCAO/r group, might due to the endogenous recovery after stroke, which has been further significantly enhanced by iTBS, as evidenced by the improved number of EdU^+^ cells in DG (*P* < 0.01) and SVZ (*P* < 0.01). Transcriptome-wide RNA sequencing technology followed by further bioinformatics analysis was performed to explore the mechanism of iTBS protecting against cerebral ischemia-induced neuronal damage. We found 1157 DEGs with 42 genes up-regulated, 1115 genes down-regulated, and 1297 differentially transcripts with 423 transcripts up-regulated, 874 transcripts down-regulated between MCAO/r and iTBS groups, respectively (Fig. 3F–I). We further used GO annotation analysis of DEGs to characterize their respective biological functions. Most biological-process-related genes between MCAO/r and iTBS groups were annotated with GO terms associated with “immune system process”, “innate immune response”, and “inflammatory response” (Fig. 3J), suggesting that iTBS protects against cerebral ischemia-induced neuronal damage probably through the anti-inflammatory mechanism.

### The spatiotemporal patterns of apoptosis and pyroptosis in the early phase of stroke

**Fig. 4:**
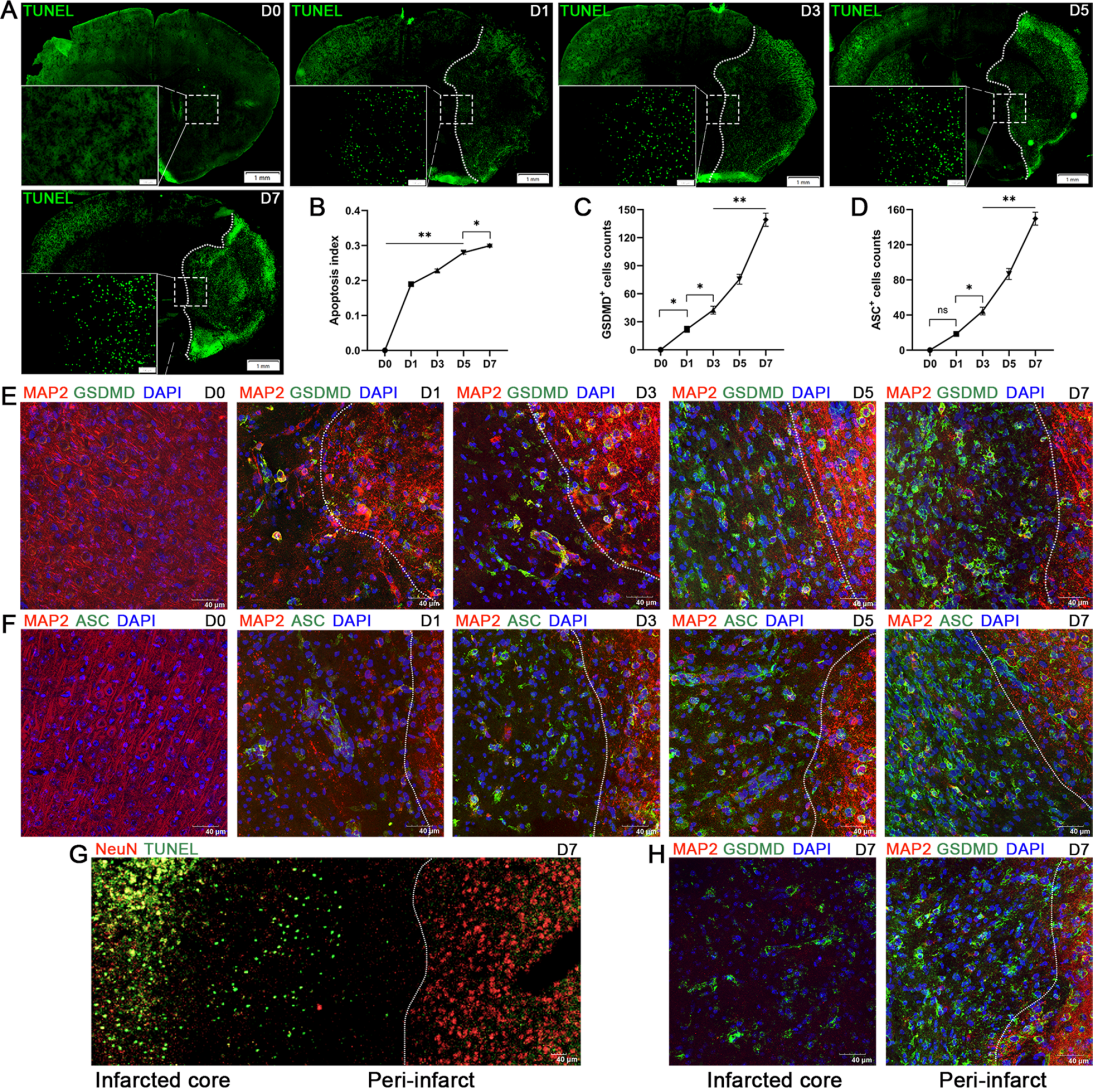
Spatiotemporal patterns of apoptosis and pyroptosis in the early phase of stroke. **A** TUNEL staining showed cell apoptosis (green) in the peri-infarcted area before stroke (D0) and at 1 day (D1), 3 days (D3), 5 days (D5), 7 days (D7) after stroke, respectively. Scale bar = 1 mm. **(B)** Quantitative analysis showed that the apoptosis index robustly increased at D1, and slightly increased from D1 to D7. Quantitative analysis showed that the number of GSDMD positive cells **(C)** slightly increased and the number of ASC positive cells **(D)** non-significantly increased between D0 and D1. Subsequently, both the number of GSDMD and ASC positive cells remarkably increased from D1 to D7. Immunofluorescence staining for MAP2/GSDMD (MAP2-red and GSDMD-green) **(E)** and MAP2/ASC (MAP2-red and ASC-green) **(F)** colocalization showed the neuronal pyroptosis in peri-infarcted area labeled with MAP2. Scale bar = 40 μm. The spatial patterns between pyroptosis and apoptosis was showed by TUNEL staining **(G)** and MAP2/GSDMD double staining **(H)**. Values are expressed as the mean ± SEM of the mean (*n* = 4). Non-significant (ns), *P < 0.05, **P < 0.01 as determined by one-way ANOVA (Tukey’s multiple comparison test)

Different from apoptosis and necrosis, pyroptosis is emerging as a common innate immune effector mechanism in vertebrates [32]. Innate immunity requires the pattern recognition receptors (PRR) to detect conserved microbial products or endogenous hazards. The activation of PRR, especially the PPR in cytosol, can not only induce the transcription of cytokines, but also induce pyroptosis, resulting in a strong inflammatory response [5]. To explore the spatiotemporal patterns of pyroptosis and apoptosis following stroke, we performed TUNEL staining and GSDMD, ASC staining before (D0) and 1 (D1), 3 (D3), 5 (D5) and 7 days (D7) after cerebral ischemia, respectively. TUNEL staining detected DNA fragmentation represents a characteristic of late stage apoptosis or necrosis [33]. The apoptosis index in peri-infarcted area at D1 displayed a robust increase compared with that at D0, and further slightly increased over a time course from D1 to D7 after cerebral I/R injury with smaller growth rates (D0, 0.00 ± 0.00, D1, 0.19 ± 0.004, D3, 0.23 ± 0.004, D5, 0.28 ± 0.004, D7, 0.30 ± 0.004; Fig. 4A, B). The GSDMD cleavage and membrane pores formation in pyroptosis are the most characterized event distinguished from other kinds of regulated cell death [5]. In contrast, confocal images for MAP2/GSDMD and MAP2/ASC colocalization analysis showed a low but significant increase in the number of GSDMD positive cells (D0, 0.00 ± 0.00, D1, 22.00 ± 3.37; *P* < 0.05) and non-significant difference in ASC positive cells (D0, 0.00 ± 0.00; D1, 18.50 ± 2.47; *P* = 0.11) between D0 and D1 in the peri-infarcted area labeled with MAP2, a sensitive indicator for the assessment of neuronal injury [34]. Subsequently, both the number of GSDMD and ASC positive cells remarkably increased over a time course from D1 to D7 after cerebral I/R injury with higher growth rates (GSDMD: D3, 42.50 ± 4.17, D5, 75.50 ± 5.30, D7, 139.30 ± 7.09; ASC: D3, 44.50 ± 4.33, D5, 86.75 ± 6.30, D7, 149.80 ± 7.43; Fig. 4C–F). Our work confirms that the peak of pyroptosis is later than that of apoptosis in the peri-infarcted area. Moreover, we also originally report that the duration and severity of pyroptosis are more persistent and severe than that of apoptosis during the early course of stroke, suggesting that timely intervention to inhibit pyroptosis following stroke is potentially valuable, and iTBS is more likely to protect against cerebral ischemia induced neuronal damage by inhibiting pyroptosis rather than apoptosis from acute phase (1–7 days) to early subacute phase (7 days until 3 months) [35]. Furthermore, in addition to the differences in temporal patterns, we also found different spatial patterns between pyroptosis and apoptosis. TUNEL positive cells were mainly located in the infarcted core (Fig. 4G), while GSDMD positive cells were more distributed in the peri-infarcted area at the 7th day post-stroke (Fig. 4H).

### ITBS improved the local inflammatory microenvironment and inhibited inflammasome-induced neuronal pyroptosis

**Fig. 5:**
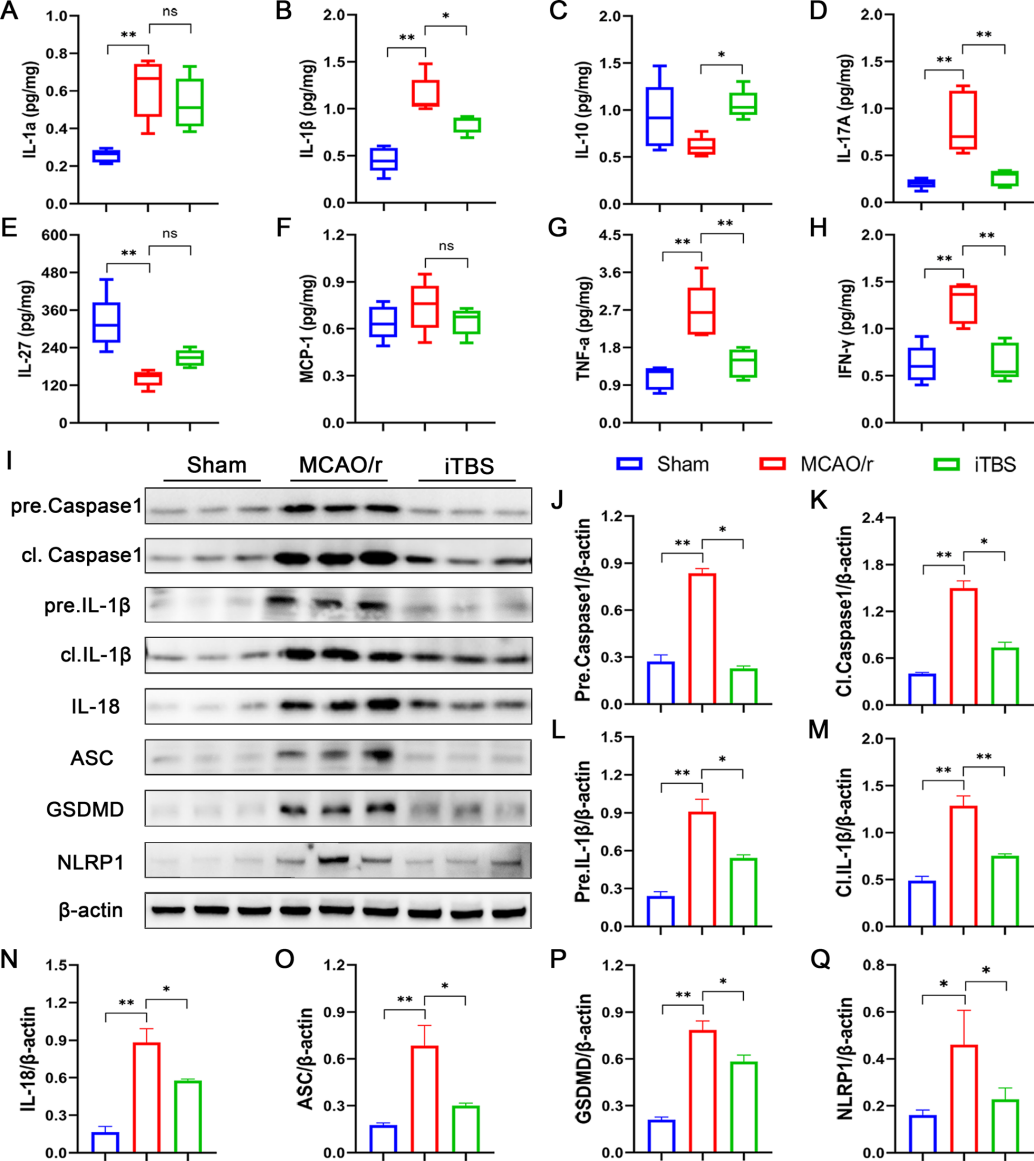
ITBS improved local inflammatory status and down-regulated the expression of proteins associated with neuronal pyroptosis. Multiplex cytokine bioassays profiled the levels of 8 available cytokines, including IL-1α **(A)**, IL-1β **(B)**, IL-10 **(C)**, IL-17A **(D)**, IL-27 **(E)**, MCP-1 **(F)**, TNF-α **(G)** and IFN-γ **(H)**. ITBS significantly ameliorated the high levels of IL-1β, IL-17A, TNF-α, IFN-γ in MCAO/r group and elevated the level of IL-10 (*n* = 5). Values are expressed as the mean and 95% confidence interval. Representative bands of the western blot **(I)** and quantitative analysis of pre.Caspase1 **(J)**, cl.Caspase1 **(K)**, pre.IL-1β **(L)**, cl.IL-1β **(M)**, IL-18 **(N)**, ASC **(O)**, GSDMD **(P)** and NLRP1 **(Q)** normalized to β-actin (*n* = 6). ITBS ameliorated cerebral ischemia induced increases in the expression of proteins above. Values are expressed as the mean ± SEM of the mean. Non-significant (ns), *P < 0.05, **P < 0.01 as determined by one-way ANOVA (Tukey’s multiple comparison test)

We profiled the levels of 13 cytokines, using the bead-based immunoassay LEGENDplex, to explore the local inflammatory status of infarcted tissue. Notably, 5 out of 13 cytokines were below the detection limit (data not shown). Five pro-inflammatory cytokines, including IL-1α (Sham, 0.26 ± 0.02, MCAO/r, 0.62 ± 0.07; *P* < 0.01; Fig. 5A), IL-1β (Sham, 0.46 ± 0.06, MCAO/r, 1.14 ± 0.09; *P* < 0.01; Fig. 5B), IL-17A (Sham, 0.20 ± 0.02, MCAO/r, 0.84 ± 0.15; *P* < 0.01; Fig. 5D), tumor necrosis factor alpha (TNF-α) (Sham, 1.08 ± 0.12, MCAO/r, 2.67 ± 0.29; *P* < 0.01; Fig. 5G) and interferon-γ (IFN-γ) (Sham, 0.62 ± 0.09, MCAO/r, 1.28 ± 0.10; *P* < 0.01; Fig. 5H), which were significantly elevated in the MCAO/r group compared with sham-operated mice. ITBS significantly reduced the levels of IL-1β (iTBS, 0.84 ± 0.04; *P* < 0.05; Fig. 5B), IL-17A (iTBS, 0.26 ± 0.04; *P* < 0.01; Fig. 5D), TNF-α (iTBS, 1.43 ± 0.15; *P* < 0.01; Fig. 5G), IFN-γ (iTBS, 0.64 ± 0.09; *P* < 0.01; Fig. 5H) and elevated the level of anti-inflammatory cytokine IL-10 (MCAO/r, 0.61 ± 0.05, iTBS, 1.06 ± 0.07; *P* < 0.05; Fig. 5C) compared with MCAO/r group.

Inflammasome (e.g., NLRP1, ASC, AIM2) and caspase protein family (e.g., Caspase1) play key roles in regulating pyroptosis [5]. In comparison with Sham group, western blotting revealed that the expression of inflammasome associated proteins [i.e., NLRP1 (*P* < 0.05), ASC, and cl.Caspase1 (*P* < 0.01)] and pyroptosis associated proteins [i.e., GSDMD, cl.IL-1β, and IL-18 (*P* < 0.01)] were remarkably elevated after MCAO/r (Fig. 5I–Q). Not only precursors but also activated fragments of pro-inflammatory Caspase1, IL-1β and IL-18 increased in cerebral I/R condition (*P* < 0.01). Notably, iTBS significantly mitigated the levels of inflammasome components and pyroptosis-associated proteins (*P* < 0.05) (Fig. 5I–Q).

**Fig. 6:**
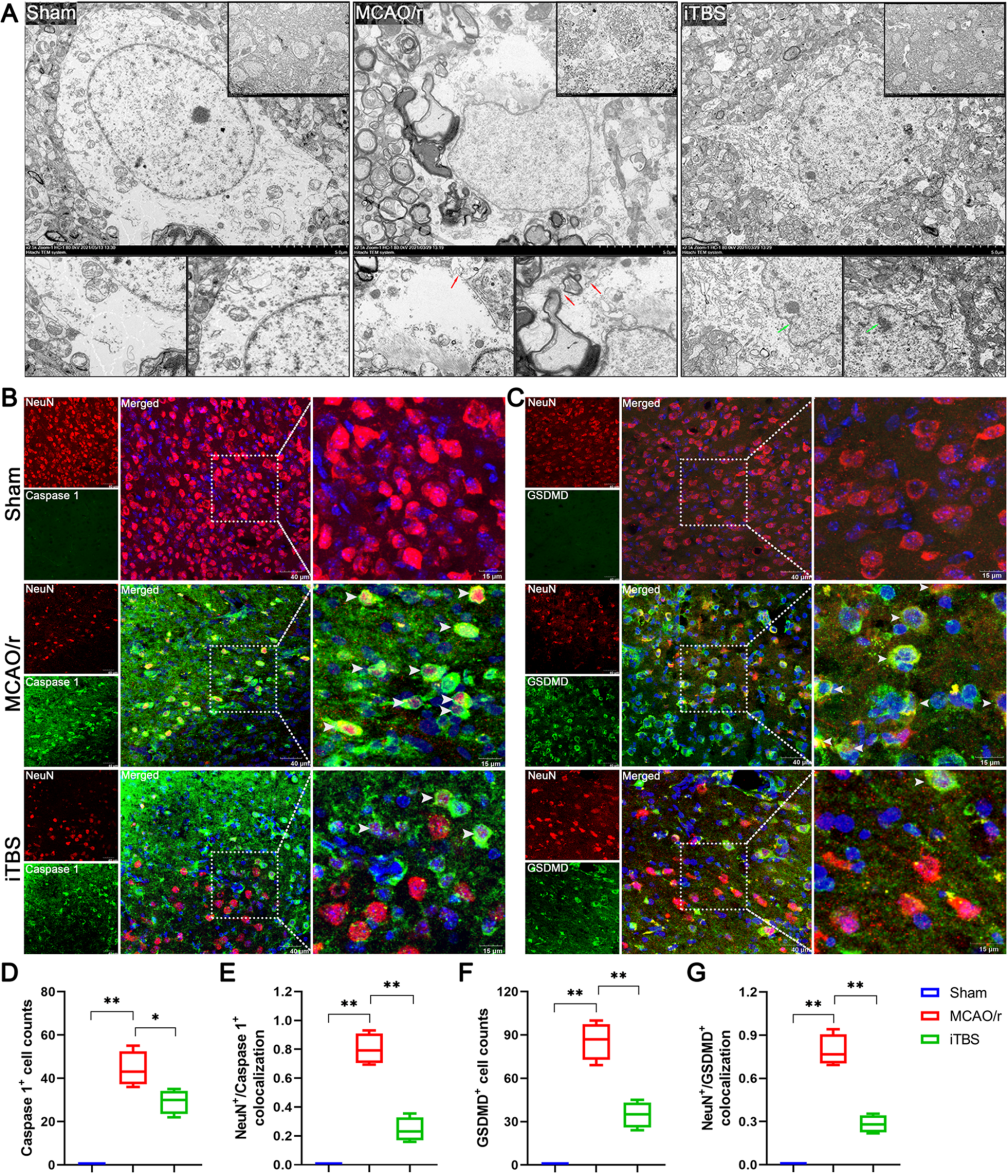
ITBS reduced the number of pyroptotic cells and proteins associated with pyroptosis on neurons. **A** Ultrastructural comparison by electron microscopy among Sham, MCAO/r and iTBS groups (*n* = 3). Cell membrane pores (Red arrow) formed in ischemic neuron of MCAO/r group, where cell nuclear membrane was complete, but chromatin disappeared. Cell nuclear membrane in iTBS group showed a concave shape, and chromatin condensed (Green arrow). Immunofluorescence staining for NeuN/Caspase1 colocalization (NeuN-red and Caspase1-green) **(B)**, NeuN/GSDMD colocalization (NeuN-red and GSDMD-green) **(C)** showed the location of pyroptotic cells among Sham, MCAO/r and iTBS groups. Scale bar = 40 μm. Quantitative analysis of cell counts showed that iTBS significantly reduced the number of Caspase1 **(D)** and GSDMD **(F)** positive cells in peri-infarcted area. Colocalization analysis revealed that iTBS significantly reduced the expression of Caspase1 **(E)** and GSDMD **(G)** on neurons in the peri-infarcted area compared with MCAO/r group. Values are expressed as the mean and 95% confidence interval (*n* = 4). *P < 0.05, **P < 0.01 as determined by one-way ANOVA (Tukey's multiple comparison test)

Transmission electron microscopy showed that the morphological changes of neuron in the Sham group were normal with complete cell membrane, nuclear membrane. By contrast, cell membrane pores formed and lost integrity in ischemic neuron of MCAO/r group, where cell nuclear membrane was complete, but chromatin disappeared. Most organelles were no longer recognizable in pyroptotic neuron. The neuron in iTBS group displayed a relatively intact morphology compared with MCAO/r group, where cell membrane was complete. However, the cell nuclear membrane in iTBS group also showed a concave shape, and chromatin condensed (Fig. 6A).

Immunofluorescence staining for NeuN/Caspase1 (Fig. 6B), NeuN/GSDMD (Fig. 6C) and NeuN/IL-1β (Additional file 1: Fig. S2A) colocalization in the peri-infarcted area showed the cellular localization of pyroptotic cells after ischemia/reperfusion injury. Similar to the results of Western blot, iTBS significantly reduced the number of Caspase1 (*P* < 0.05; Fig. 6D), GSDMD (*P* < 0.01; Fig. 6F) and IL-1β (*P* < 0.05; Additional file 1: Fig. S2B) positive cells in the peri-infarcted area. Colocalization analysis revealed that iTBS significantly reduced the expression of Caspase1, GSDMD and IL-1β on neurons in the peri-infarcted area compared with MCAO/r group (*P* < 0.01; Fig. 6E, G; Additional file 1: Fig. S2C).

### ITBS inhibited neuronal pyroptosis in the peri-infarcted area rather than at the border of infarcted core

**Fig. 7:**
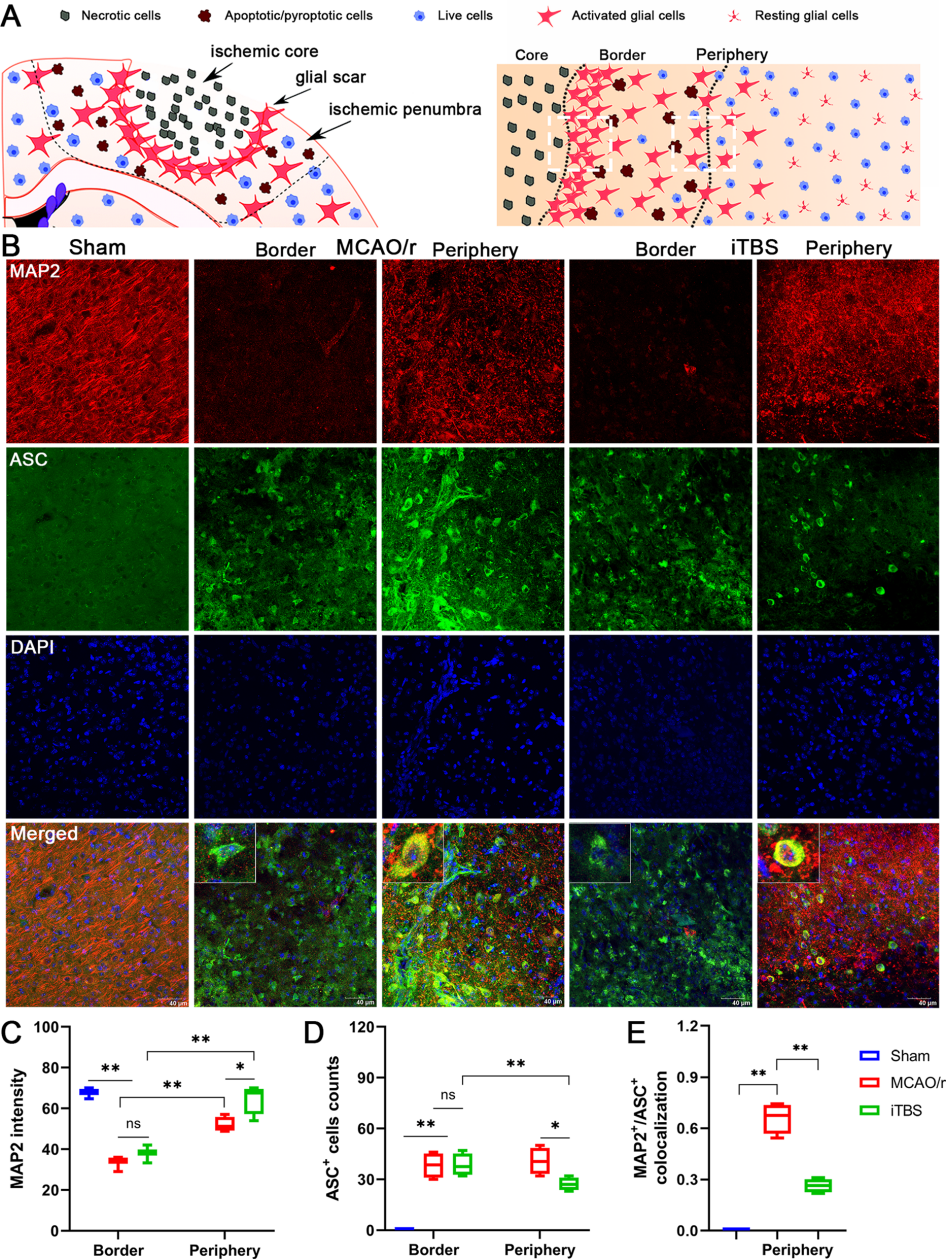
ITBS inhibited expression of ASC in the peri-infarcted area. **A** Schematic representation of the distribution of necrotic cells, apoptotic/pyroptotic cells and activated glial cells in the different regions of the ischemic hemisphere, including ischemic core and peri-infarcted area. **B** Immunofluorescence staining for MAP2/ASC colocalization (MAP2-red and ASC-green) showed the location of pyroptotic cells among Sham, MCAO/r and iTBS groups. Scale bar = 40 μm. **C** Quantitative analysis of MAP2 fluorescence intensity showed that iTBS enhanced the expression of MAP2 in the peri-infarcted area. **D** ITBS significantly reduced the number of ASC positive cells in peri-infarcted area rather than at the border of infarcted core. **E** MAP2/ASC colocalization analysis revealed that iTBS significantly reduced the expression of ASC on neurons in the peri-infarcted area compared with MCAO/r group. Values are expressed as the mean and 95% confidence interval (*n* = 4). Non-significant (ns), *P < 0.05, **P < 0.01 as determined by one-way ANOVA (Tukey’s multiple comparison test) and two-way ANOVA (Bonferroni's multiple comparison test)

**Fig. 8:**
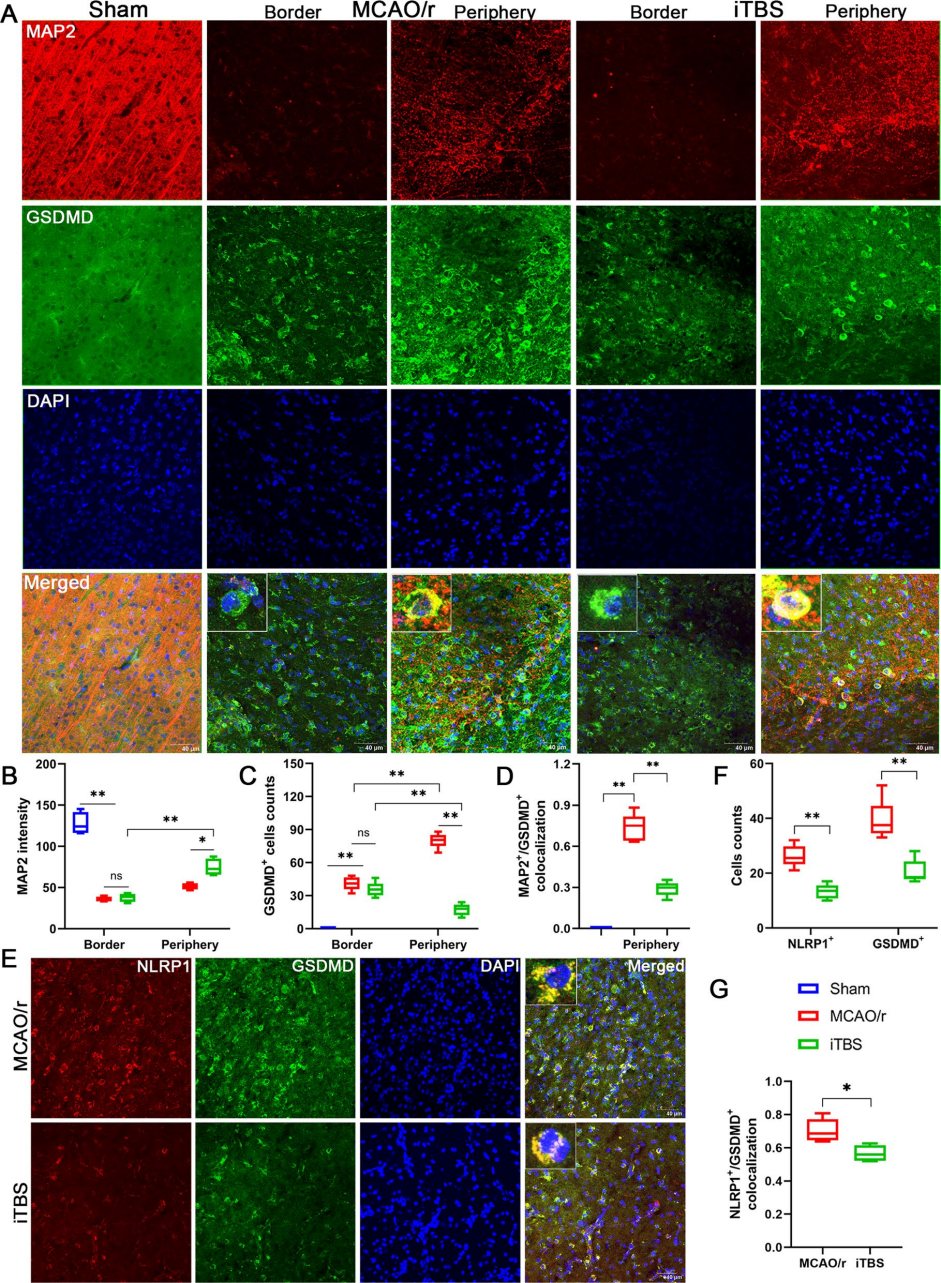
ITBS inhibited expression of GSDMD and NLRP1 in the peri-infarcted area. **A** Immunofluorescence staining for MAP2/GSDMD colocalization (MAP2-red and GSDMD-green) showed the location of pyroptotic cells among Sham, MCAO/r and iTBS groups. Scale bar = 40 μm. **B** Quantitative analysis of MAP2 fluorescence intensity showed that iTBS enhanced the expression of MAP2 in the peri-infarcted area. **C** High number of GSDMD positive cells were located in the peri-infarcted area than that at the border of infarcted core in MCAO/r group, while iTBS significantly reduced the number of GSDMD positive cells in peri-infarcted area. **D** MAP2/GSDMD colocalization analysis revealed that iTBS significantly reduced the expression of GSDMD on neurons in the peri-infarcted area compared with MCAO/r group. Values are expressed as the mean and 95% confidence interval (*n* = 4). Non-significant (ns), *P < 0.05, **P < 0.01 as determined by one-way ANOVA (Tukey’s multiple comparison test) and two-way ANOVA (Bonferroni’s multiple comparison test). Immunofluorescence staining for NLRP1/GSDMD colocalization (NLRP1-red and GSDMD-green) **(E)** and quantitative analysis **(F, G)** showed iTBS reduced the expression of NLRP1 in GSDMD positive cells in the peri-infarcted area (*n* = 4). Scale bar = 40 μm. Values are expressed as the mean and 95% confidence interval (*n* = 4). *P < 0.05, **P < 0.01 as determined by unpaired Student’s *t* test

MAP2 protein is a family of heat-stable, phosphoproteins expressed predominantly in the cell body and dendrites of neurons. MAP2 proteins are abundantly expressed in neurons, which is frequently used as a neuronal or dendritic marker, because it is present in the cell body and dendrites of neurons but absent in axons [34]. Confocal microscopy and quantitative analysis showed that compared with Sham group, mice in the MCAO/r group had essentially few MAP2 positive fluorescence signal at the border of infarcted core (*P* < 0.01). Furthermore, less MAP2 fluorescence intensity and greater MAP2 dispersion were found in the peri-infarcted area, reflecting dendritic damage to the peri-infarcted neurons. In contrast, compared with MCAO/r group, the MAP2 fluorescence intensity significantly increased (*P* < 0.05) and dendritic morphology of cortical neurons in the peri-infarcted area was slightly repaired in iTBS group. However, there was few and non-significant difference in MAP2 fluorescence signal at the border of infarcted core (*P* = 0.95) (Figs. 7B, C, 8A, B). These data indicate that iTBS significantly inhibits cerebral I/R injury induced synaptic damage and neuronal degeneration in the peri-infarcted area.

Similar to the MAP2, immunofluorescence staining for ASC and GSDMD showed that cerebral ischemic mice in the MCAO/r group had higher number of GSDMD positive cells (*P* < 0.01) but not ASC positive cells (*P* > 0.05) located in the peri-infarcted area than that at the border of infarcted core. ITBS significantly reduced the number of both ASC (*P* < 0.05) and GSDMD positive cells (*P* < 0.01) in the peri-infarcted area, while there were no significant differences at the border of infarcted core between iTBS and MCAO/r groups (*P* > *0.05*) (Figs. 7B, D, 8A, C). Colocalization analysis revealed that iTBS significantly reduced the expression of ASC and GSDMD on neurons in the peri-infarcted area compared with MCAO/r group (*P* < 0.01) (Figs. 7E, 8D). To further analyze the pyroptosis of neuron in peri-infarcted area by counting NLRP1 positive cells, the inflammasome mainly presented in motor neurons [36], we found that the increased number of NLRP1 positive cells induced by cerebral I/R injury was significantly alleviated by iTBS (*P* < 0.01; Fig. 8E, F). On the other hand, immunofluorescence staining for NLRP1/GSDMD colocalization showed that iTBS also significantly decreased percentage of NLRP1^+^/GSDMD^+^ cell (*P* < 0.05; Fig. 8G). Taken together, the results strongly support that iTBS post-treatment is capable of inhibiting cerebral ischemia-induced neuronal pyroptosis in the peri-infarcted area rather than at the border of infarcted core.

### The anti-inflammatory effect of iTBS might be mediated by modulating microglial phenotypes via inhibiting TLR4/NFκB/NLRP3 signaling pathway

**Fig. 9:**
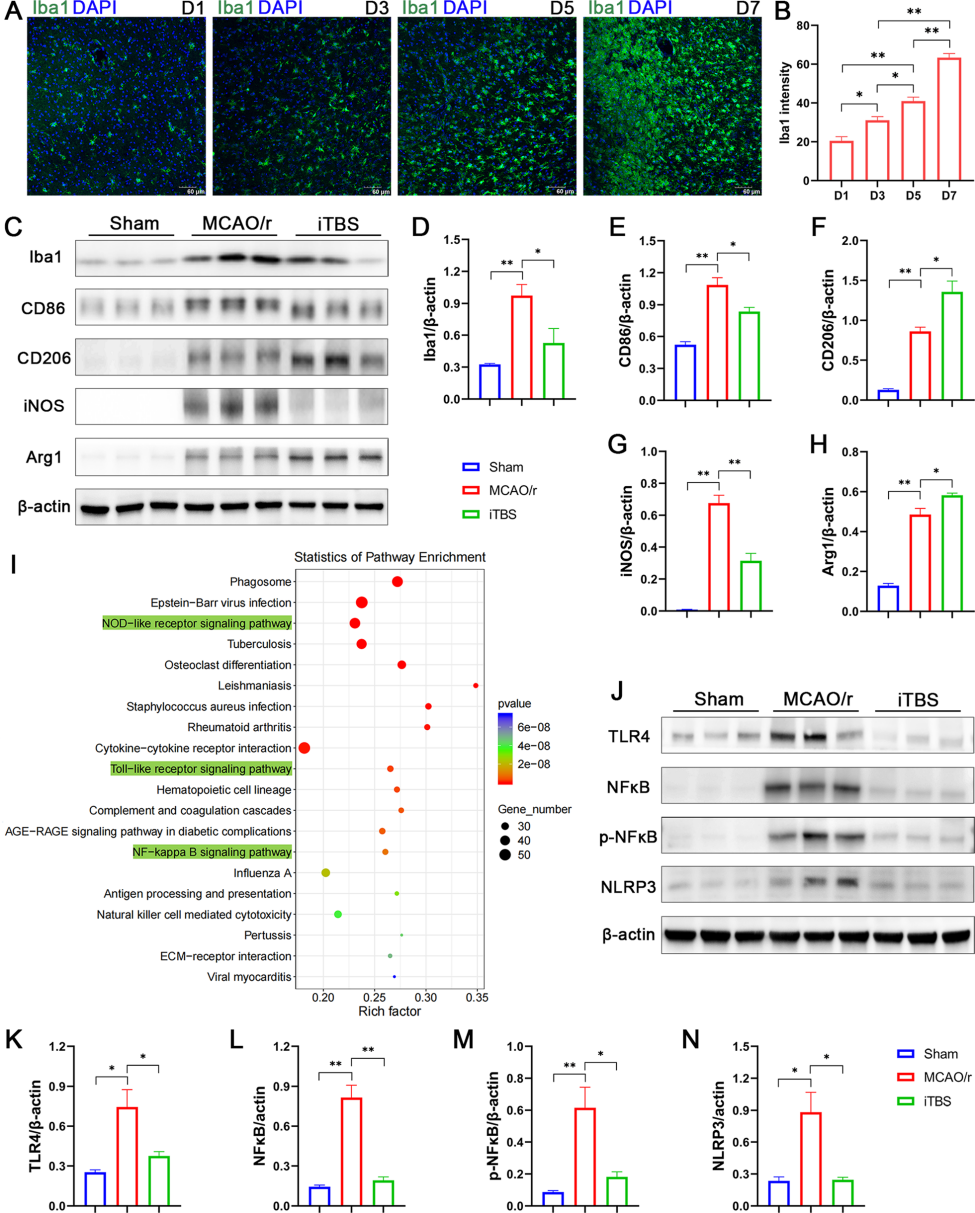
ITBS modulated microglial phenotypes via inhibiting TLR4/NFκB/NLRP3 signaling pathway. Immunofluorescence staining for Iba1 **(A)** and quantitative analysis **(B)** showed microglial activation over a time course from D1 to D7 after cerebral I/R injury (*n* = 4). Scale bar = 60 μm. Representative bands of the western blot **(C, J)** and quantitative analysis of Iba1 **(D)**, CD86 **(E)**, CD206 **(F)**, iNOS **(G)**, Arg1 **(H)**, TLR4 **(K)**, NFκB **(L)**, p-NFκB (**M**) and NLRP3 **(N)** normalized to β-actin (*n* = 6). ITBS reduced protein levels associated with classical M1 phenotypic activation (CD86, iNOS) and elevated protein levels associated with alternative M2 phenotypic activation (CD206, Arg1), respectively. **I** KEGG pathway analysis of differentially expressed genes showed that the potential mechanism of iTBS regulating innate immune responses were highly associated with NOD-like receptor, Toll-like receptor and NFκB signaling pathways involved. ITBS also ameliorated the high levels of TLR4, NFκB**,** p-NFκB and NLRP3 reduced by cerebral ischemia. Values are expressed as the mean ± SEM of the mean. *P < 0.05, **P < 0.01 as determined by one-way ANOVA (Tukey's multiple comparison test)

Glial cells, particularly microglia, are thought to play a pivotal role in initiating and guiding innate immune responses to CNS disease. It is well known that over-activation of microglia can affect local inflammation, leading to changes in neuronal microenvironment and neuronal damage [37]. Quantitative analysis revealed that the Iba1 fluorescence intensity strongly increased from D1 to D7 after cerebral I/R injury (D1, 20.50 ± 2.10, D3, 31.13 ± 1.85, D5, 40.95 ± 2.06, D7, 63.36 ± 2.16; Fig. 9A, B). Confocal microscopy of Iba1 staining also showed that a distinct glial scar surrounding the infarcted core can be observed at D7 after cerebral ischemia, whereas it does not form during the first 5 days after stroke (Fig. 9A). Moreover, immunofluorescence staining for GFAP/TUNEL showed the activation of astrocytes over a time course from D1 to D7 after cerebral I/R injury (Additional file 1: Fig. S1F). Western blotting and quantitative analysis further showed that activity of microglia significantly increased in the MCAO/r group compared with Sham group (*P* < 0.01; Fig. 9C, D). Notably, iTBS not only significantly alleviated microglial activation, but also induced a switch in microglial M1/M2 phenotype, as evidenced by the inhibition and elevation of protein levels associated with classical M1 phenotypic activation (CD86, iNOS) and alternative M2 phenotypic activation (CD206, Arg1), respectively (*P* < 0.05; Fig. 9E–H).

We further used KEGG enrichment analysis to reveal the potential mechanism of iTBS regulating innate immune responses. The most significantly enriched pathways were: “NOD-like receptor signaling pathway”, “Toll-like receptor signaling pathway” and “NF-kappa B (NFκB) signaling pathway” (Fig. 9I). Ample evidence shows that microglia/macrophage polarization is mainly regulated by toll-like receptors (TLRs) pathway, which also plays a critical role in the innate immune responses [38]. To further delineate the mechanisms potentially involved in the modulating of microglial phenotypes after iTBS, we found that the expression of TLR4 (*P* < 0.05; Fig. 9K), NFκB (*P* < 0.01; Fig. 9L), p-NFκB (*P* < 0.01; Fig. 9M), NLRP3 (*P* < 0.05; Fig. 9N) were significantly increased in MCAO/r group, which were significantly attenuated in iTBS group (*P* < 0.05), suggesting that TLR4/NFκB/NLRP3 signaling pathway might be a crucial regulator for modulating microglial polarization and regulating innate immune responses after iTBS treatment.

### ITBS modulated microglial phenotypes in the peri-infarcted area rather than at the border of infarcted core

**Fig. 10:**
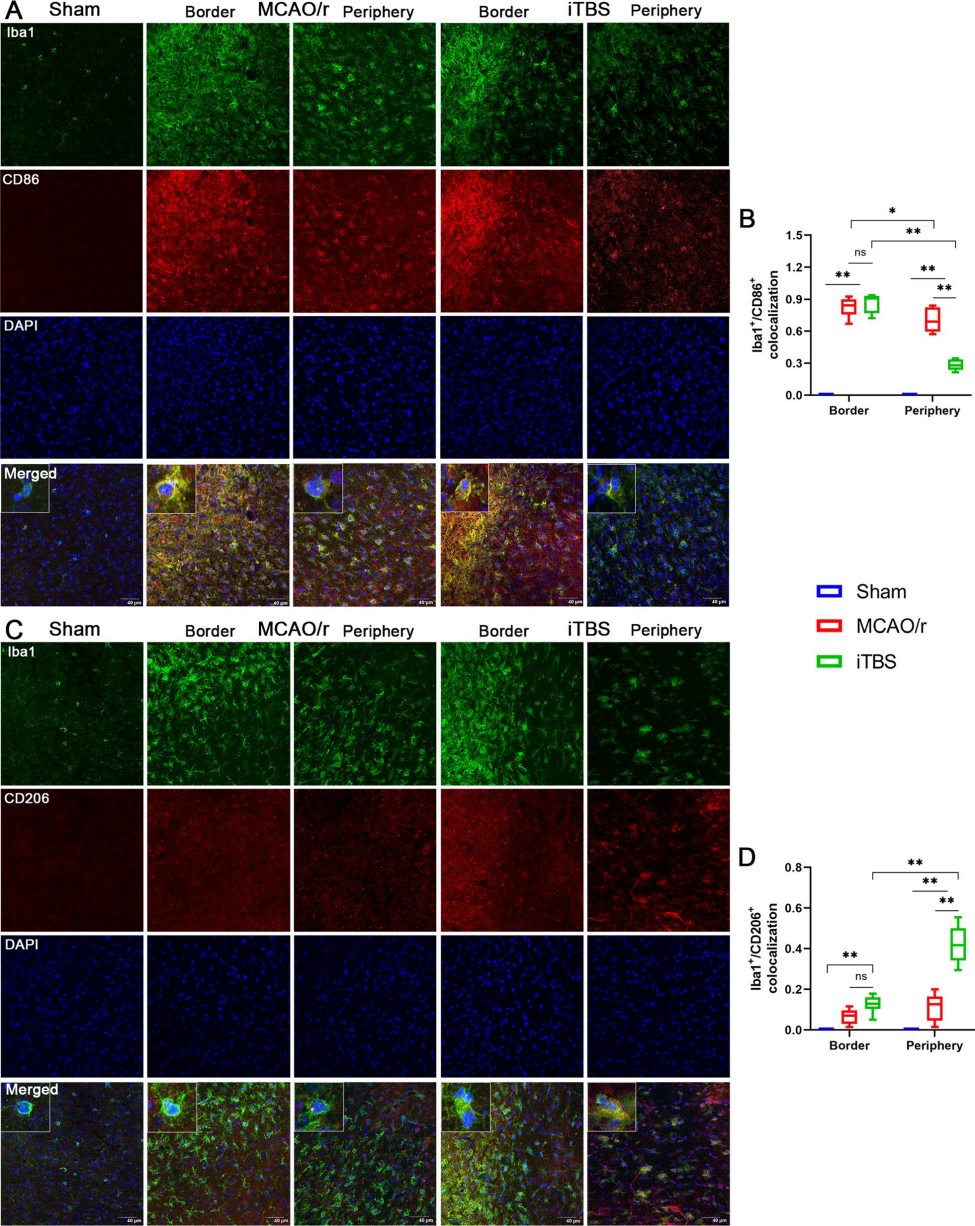
ITBS modulated microglial phenotypes in the peri-infarcted area. **A** Immunofluorescence staining for Iba1/CD86 colocalization (Iba1-green and CD86-red) showed the location of M1-polarised microglia (Iba1^+^/CD86^+^) among Sham, MCAO/r and iTBS groups. Numerous microglial cells and M1-polarised microglia abundant at the border of infarcted core. Scale bar = 40 μm. **B** Iba1/CD86 colocalization analysis revealed that iTBS significantly reduced the expression of CD86 on microglia in the peri-infarcted area compared with MCAO/r group. **C** Immunofluorescence staining for Iba1/CD206 colocalization (Iba1-green and CD206-red) showed the location of M2-polarised microglia (Iba1^+^/CD206^+^) among Sham, MCAO/r and iTBS groups. Few CD206 positive fluorescent signal was observed at the border of infarcted core in both MCAO/r and iTBS groups. Scale bar = 40 μm. **D** Iba1/CD206 colocalization analysis revealed that iTBS significantly up-regulated the expression of CD206 on microglia in the peri-infarcted area compared with MCAO/r group. Values are expressed as the mean and 95% confidence interval (*n* = 4). *P < 0.05, **P < 0.01 as determined by one-way ANOVA (Tukey’s multiple comparison test) and two-way ANOVA (Bonferroni's multiple comparison test)

Furthermore, the confocal images for Iba1/CD86 and Iba1/CD206 colocalization analysis were acquired from the border of infarcted core and peri-infarcted area. We observed numerous microglial cells and M1-polarised microglia (Iba1^+^/CD86^+^) abundant at the border of infarcted core (Fig. 10A, B). Few CD206 positive fluorescent signal was observed at the border of infarcted core in both MCAO/r and iTBS groups (Fig. 10C). Immunoactivity analysis demonstrated a marked shift towards the M1 phenotype in the peri-infarcted area which was lessened in iTBS group, as evidenced by the lower percentage of Iba1^+^/CD86^+^ microglia compared with MCAO/r group (*P* < 0.01; Fig. 10B). By contrast, iTBS mice displayed a commensurate increase in M2 phenotype activation compared with MCAO/r group, as evidenced by the improved percentage of Iba1^+^/CD206^+^ microglia in peri-infarcted area (*P* < 0.01; Fig. 10C, D). There were no significant differences in both number of M1 (*P* = 0.66) and M2-polarised microglia (*P* = 0.15) at the border of infarcted core between MCAO/r and iTBS groups. This demonstrates that iTBS significantly shifts the M1/M2 phenotype balance by curbing proinflammatory M1 activation and enhancing the anti-inflammatory M2 activation in peri-infarcted area rather than at the border of infarcted core during the course of stroke, which may contribute to inhibiting the pyroptosis-associated inflammatory response in cortical neuron.

### Depletion of microglia eliminated the motor functional improvements after iTBS treatment

**Fig. 11:**
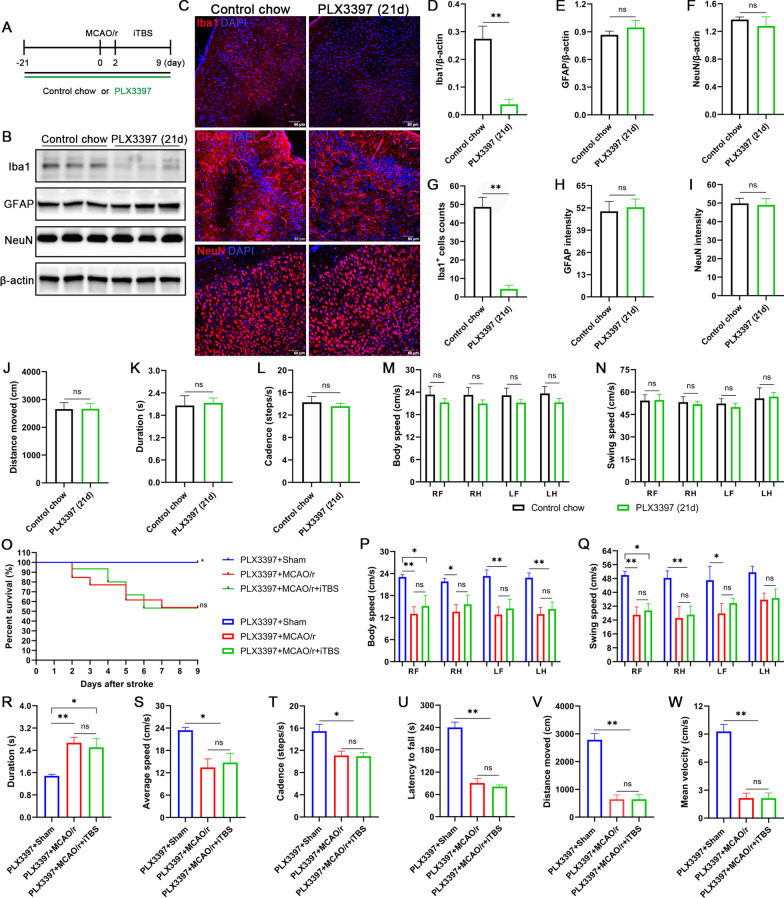
Depletion of microglia eliminated locomotor recovery of MCAO/r injury mice after iTBS treatment. **A** Mice received the CSF1R inhibitor PLX3397 for 3 weeks prior to induction of MCAO/r and the diet was maintained until the end of 1-week iTBS treatment. Representative bands of the western blot **(B)** and quantitative analysis of Iba1 **(D)**, GFAP **(E)**, NeuN **(F)**. Immunofluorescence staining **(C)** and quantitative analysis of Iba1 positive cells counts **(D)**, GFAP intensity **(H)**, and NeuN intensity **(I)**. Scale bar = 60 μm. Values are expressed as the mean ± SEM of the mean (*n* = 4). Non-significant (ns), *P < 0.05, **P < 0.01 as determined by unpaired Student’s t test. 21-day PLX3397 treatment had also no significant impact on distance move **(J)** in open filed test, duration **(K)**, cadence **(L)**, body speed **(M)**, swing speed **(N)** in CatWalk gait analysis. **(O)** Survival curve among PLX3397 + Sham, PLX3397 + MCAO/r and PLX3397 + MCAO/r + iTBS groups during 8-day observation period. Non-significant (ns), *P < 0.05 as determined by Log-rank (Mantel–Cox) test. No statistically significant differences in body speed **(P)**, swing speed **(Q)**, duration **(R)**, average speed **(S)**, cadence **(T)**, latency to fall **(U)**, distance moved **(V)** and mean velocity **(W)** were observed between PLX3397 + MCAO/r and PLX3397 + MCAO/r + iTBS groups at the end of experiment. Values are expressed as the mean ± SEM of the mean (*n* = 6). Non-significant (ns), *P < 0.05, **P < 0.01 as determined by one-way ANOVA (Tukey’s multiple comparison test). *RF* right forelimb, *RH* right hindlimb, *LF* left forelimb, *LH* left hindlimb

The CSF1R is a crucial receptor for microglial survival, and CSF1R inhibitor offers a non-invasive method for depletion of microglia without cognitive/behavioral impairments and blood–brain barrier damage [39]. As expected, 21-day PLX3397 treatment significantly decreased the expression of Iba1 (*P* < 0.01; Fig. 11B–D, G). No significant changes in markers glial fibrillary acidic protein (GFAP) (*P* > 0.05; Fig. 11B, C, E, H) and NeuN (*P* > 0.05; Fig. 11B, C, F, I) were observed. PLX3397 treatment had also no significant impact on motor behavior evaluated by open field test (*P* > 0.05; Fig. 11J, Additional file 1: Fig. S2D) and CatWalk gait analysis (*P* > 0.05; Fig. 11K–N, Additional file 1: Fig. S2E–G) from healthy mice before the induction of MCAO/r surgery. We next investigated the effect of microglia depletion in cerebral I/R injury context. Depletion of microglia aggravated the mortality rate of MCAO/r injury mice, reaching 46% in the PLX3397 + MCAO/r group. There was also no statistically significant difference in mortality rate between PLX3397 + MCAO/r and PLX3397 + MCAO/r + iTBS groups (*P* = 0.94; Fig. 11O). In the functional tests, after depletion of microglia, iTBS treatment failed to ameliorate the MCAO/r injury induced decreases (*P* > 0.05) in kinetic parameters [body speed (Fig. 11P), swing speed (Fig. 11Q), average speed (Fig. 11S), cadence (Fig. 11T)] and increase (*P* > 0.05) in temporal parameters [duration (Fig. 11R)]. Similarly, no significant differences (*P* > 0.05) were observed between PLX3397 + MCAO/r and PLX3397 + MCAO/r + iTBS groups in latency to fall (Fig. 11U), distance moved (Fig. 11V) and mean velocity (Fig. 11W) during rotarod test and open field test, respectively.

## Discussion

The current study investigated the effects of intermittent theta-burst rTMS (iTBS) on motor function and protective properties against ischemia-induced neuronal damage around the peri-infarcted area in a pre-clinical mouse model of middle cerebral artery occlusion/reperfusion. Our work revealed the spatiotemporal patterns of neuronal pyroptosis between the infarcted core and peri-infarcted area in the early phase of stroke. We originally provided evidence that iTBS applied over the ipsilesional hemisphere in the acute stage significantly ameliorated motor behavioral deficits, and inhibited cerebral ischemia-induced pyroptosis of neuron in peri-infarcted area. In addition, RNA-seq analysis and further studies revealed that the underlying mechanism of neuroprotective and functional improvement after iTBS was closely related to the modulation of microglial activation by shifting the M1/M2 phenotype balance in peri-infarcted area during the course of stroke via inhibiting TLR4/NFκB/NLRP3 signaling pathway, which may contribute to preventing the pyroptosis associated inflammatory response. Noteworthy, depletion of microglia eliminated the motor functional improvements after iTBS treatment. It is expected that these data will provide novel insights into the mechanisms of rTMS protecting against cerebral I/R injury and potential targets underlying neuronal pyroptosis in the early phase of stroke.

Therapeutic strategies for both promotion of motor recovery and protection of neurons during the early course of stroke are believed to be critical in rehabilitation treatment [40]. rTMS as a novel non-invasive neuromodulation technique has been widely used for diagnostic and treatment in stroke patients with neurological and psychiatric disorders, and has produced a wide variety of positive outcomes in acute stroke [41, 42]. A randomized controlled study reported that high frequency rTMS (10 Hz) applied over the lesional hemisphere in the early phase of stroke was more beneficial for motor improvement of the affected upper limb than those treated with low frequency rTMS (1 Hz) and sham stimulation without any adverse effect [41]. Similarly, our data also indicated that iTBS applied over the ipsilesional hemisphere in the acute stage induced significant improvements of motor strength, coordination and walking competency, as evidenced by the increased time to fall in rotarod test, inverted wire mesh grid grip test and reduced laterality index in the cylinder test, improved gait parameters in CatWalk XT gait test. There was no statistically significant difference in mortality data observed between MCAO/r and iTBS groups at the end of experiment, suggesting that iTBS applied over the lesional hemisphere in the early phase is safe if applied by optimal protocol and criteria. Mounting evidences confirmed the important and complex role of inflammation response in predicting stroke outcome and prognosis in the early stage [43]. Although several therapeutic approaches targeting excitotoxicity and early inflammatory process exhibited promising effects in ischemic stroke animal models, there were no expected results exhibited in subsequent clinical trials [2, 44]. Thus, deeper understanding of neuroinflammation and new therapies aiming to restore brain homeostasis are urgently necessary. Recent findings have shown that the potential of rTMS as non-pharmacological approach targeting anti-apoptosis and anti-inflammation, common players in several CNS diseases, such as stroke [13], traumatic brain injury [45], and spinal cord injury [46]. For example, deep high-frequency (gamma Hz) TBS has been reported to alleviate cuprizone induced neuropathologic, microglial activation and pro-inflammatory cytokine expression in the brain regions of corpus callosum, caudate putamen and cerebral cortex [47]. A study explored the mechanism of rTMS intervention applied at 4 days after cerebral ischemia on functional improvement suggested that rTMS affected penumbra tissue by reducing apoptosis in the peri-lesional area rather than with promoting neural plasticity [13].

Although both apoptosis and pyroptosis belong to a subtype of programmed cell death, and they share a lot of similarities in morphology and mechanism [48], there are few studies clarifying the effect of rTMS on pyroptosis. Indeed, during the early phases of reperfusion, expression of the inflammasome is negligible. However, the activity of inflammasome components significantly increased in the peri-infarcted area which was exposed to sublethal injury and pro-inflammatory stimuli by damage-associated molecular patterns (DAMPs) released by the neighboring necrotic cells in infarcted core [49]. As with the results presented in our study, few MAP2 positive and less GSDMD positive fluorescent signal were observed at the border of infarcted core, probably because the neurons here were already necrotic or apoptotic due to the large number of toxic signals received, as evidenced by more TUNEL positive cells distributed in the infarcted core. In contrast, large amount of GSDMD positive cells were abundant in peri-infarcted area labeled with MAP2 and significantly increased over a time course from 1 to 7 days after cerebral I/R injury. When expression of inflammasome proteins in the peri-infarcted area markedly upregulated and accumulated, the size of infarcted area continues to expand through pyroptosis over several days after reperfusion [49]. Our results of TUNEL staining also provided evidence that the peak of neuronal pyroptosis was later than that of apoptosis in the peri-infarcted area during early phase of stroke, suggesting that timely intervention to inhibit neuronal pyroptosis following stroke is potentially valuable. In general, numerous studies indicated that inflammasome activation and pyroptosis would aggravate ischemic injury, while inhibiting inflammasome activation has been reported to exert neuroprotection effects. For example, inflammasome associated proteins (NLRP1, ASC and Caspase1) were acutely elevated in the cerebrospinal fluid of patients suffering from traumatic brain injury, and the degree of elevation significantly correlated with long-term functional outcome [50]. In contrast, deletion or inhibition of the inflammasome components (NLRP3, ASC or Caspase1) could reduce infarcted size [51, 52]. Our study originally demonstrated that iTBS applied over the ipsilesional hemisphere in the acute stage significantly reduced the expression of inflammasome associated proteins (i.e., NLRP1, ASC and cl.Caspase1) and pyroptosis-associated proteins (i.e., GSDMD, cl.IL-1β and IL-18). Double immunofluorescence staining for NeuN/Caspase1, NeuN/GSDMD, NeuN/IL-1β, MAP2/ASC, MAP2/GSDMD and NLRP1/GSDMD further confirmed that iTBS post-treatment is capable of inhibiting cerebral ischemia-induced pyroptosis of neuron in peri-infarcted area rather than at the border of infarcted core.

Microglia, as the resident macrophage of CNS, are early contributors to neuroinflammation and the first line of defense against ischemic stroke [53]. Our results confirmed that the levels of IL-1β and IL-18 were significantly upregulated after cerebral ischemia. Recently, studies showed that the membrane pore formed during GSDMD-executed pyroptosis serve as a gate for extracellular secretion of mature IL-1β and IL-18 [5]. Moreover, M1-like microglia are also a prominent source of pro-inflammatory cytokines, such as IL-1β, IL-18, IL-6, TNF-α, and IFN-γ, as well as neurotoxic mediators, such as nitric oxide (NO), reactive oxygen species (ROS), matrix metalloproteinase (MMP), prostaglandin (PG) E2. In contrast, M2 polarized microglia (alternative activation) are mainly involved in the phagocytosis and cleaning of the injured site, involved in tissue repair and extracellular matrix remodeling, and can also release anti-inflammatory cytokines, such as transforming growth factor-β (TGF-β), IL-4, IL-10 and insulin-like growth factor-1 (IGF-1), etc. [54]. Our cytokine bioassays also confirmed that IL-1α, IL-1β, IL-17A, TNF-α and IFN-γ were significantly elevated in the MCAO/r group.

Accumulating evidence suggested that non-invasive neuromodulation technique could affect microglial function. For example, Anton Pikhovych et al. suggested anodal tDCS to reduce Iba1-positive microglia in the cortex of healthy mouse [55]. In contrast, Peruzzotti-Jametti et al. reported that cathodal tDCS could down-regulate constitutive expression of Iba1 on microglia in the peri-ischemic cortex of mice when applied during acute focal cerebral ischemia [56]. Another experiment found that cathodal tDCS increased the number of iNOS-positive M1-polarized microglia without affecting CD206-positive M2-polarized microglia [57]. Moreover, a recent study demonstrated that low frequency tFUS (0.5 MHz) applied to the ischemic hemisphere of mice for 7 consecutive days significantly increased the percentage of CX3CR1/CD206^+^ cells (M2 phenotype). However, there was no significant difference in the number of CX3CR1/CD16/32^+^ cells (M1 phenotype) [17]. Like the heterogeneous results after tDCS or tFUS, the exact effect of rTMS on microglia in vivo has also been largely unexplored. Candela Zorzo et al. reported that 3 days of high-frequency rTMS applied in healthy rats does not alter microglia proliferation and inflammatory responses [58]. A recent study demonstrated that 5-min daily cTBS (3 pulses of 50 Hz repeated every 200 ms, intensity at 200 G) applied on the infarcted hemisphere beginning at 3 h after photothrombotic stroke injury for continuous 5-day reduced M1 phenotype microglial activation and suppressed pro-inflammatory cytokines production [59]. In contrast, Lukas Muri et al. reported that cTBS (three 30 Hz pulses repeated at intervals of 100 ms for 200 times) increased abundance of CD68^+^ cells (M1 phenotype) in cerebral cortex and hippocampal dentate gyrus [60]. Indeed, our present data set provided further evidence in this regard. We confirmed that iTBS (ten 50 Hz bursts with 3 pulses each repeated 20 times at 5 Hz intervals) applied over the ipsilesional hemisphere in the early phase of stroke induced a shift in M1/M2 phenotype activation. Noteworthy, depletion of microglia using CSF1R inhibitor PLX3397 eliminated the motor functional improvements after iTBS treatment. Moreover, our results also confirmed the pattern of microglial activation vary substantially between the border of infarcted core and peri-infarcted area. Pro-inflammatory microglia were abundant in the peri-infarcted area, while there were less anti-inflammatory microglial cells in both border of infarcted core and peri-infarcted area at 9th day post-stroke. ITBS significantly increased M2 phenotype activation in the peri-infarcted area, which may explain the spatiotemporal pattern of neuronal pyroptosis, namely, lower expression of GSDMD and NLRP1 on neurons in the peri-infarcted area compared with MCAO/r group. Although non-invasive neuromodulation techniques have been previously reported to regulate microglial activation, there are no studies revealing the potential mechanisms. Our RNA-seq analysis showed that the protection against cerebral I/R injury after iTBS was highly associated with the regulation of innate immune responses involving NOD-like receptor, Toll-like receptor and NFκB signaling pathways. Numerous studies have shown that toll-like receptors (TLRs) pathway, a pattern-recognition receptor for innate immune responders, plays a critical role in microglia/macrophage polarization [38]. TLR4, an important mediator in the neuroinflammatory cascade in CNS, activating the p65 subunit of downstream NFκB to promote the transcription of NLRP3 components, further regulated the release of downstream inflammatory mediators through an inflammatory response [61]. Our subsequent results also confirmed that iTBS could down-regulate the high level of TLR4, NLRP3, and phosphorylation of NFκB induced by cerebral ischemia. That may be a vital mechanism for iTBS to regulate microglial polarization and exerted an anti-inflammatory effect after cerebral ischemia.

In spite of these findings, there are some limitations to be solved in the future. First, soon after an ischemic injury, peripheral monocytes/macrophages were also recruited to the injured brain via the impaired blood brain barrier, and exhibited various phenotypes similar to resident microglia [62]. There is still a lack of a specific antibody that recognize microglia and macrophages. Second, the distribution patterns of microglia are different between the brain of rodents and human [9], which may increase the difficulty of transforming basic research into clinical practice. Third, it is noteworthy that the activation of astrocytes contributes to neural injury after brain ischemia. Our results also confirmed the activation of astrocytes over a time course from D1 to D7 after cerebral I/R injury. Similar to the results in our study, previous research found that elimination of microglia exacerbated neurological defects, and its underlying mechanism was to enhance the response of astrocytes, thus promoting the expression of various pro-inflammatory mediators (e.g., IL-1α, IL-1β, iNOS, TNF-a and IL-6) in astrocytes [63]. These findings suggest that the neuroprotective effect of microglia may result from its inhibitory action on the astrocyte response. Although PLX3397 treatment did not affect the counts of astrocytes in our study, the neuron–astrocyte crosstalk in ischemic brain injury has not been explored.

## Conclusions

Our current study provides novel insight into the safety and effectiveness of rTMS as a non-invasive neuromodulation technique. We originally demonstrate that theta burst rTMS exhibits promising therapeutic effect of protecting against neuronal pyroptosis in the peri-infarcted area rather than at the border of infarcted core. Our work also provides important new mechanistic insights into how rTMS inhibits neuronal pyroptosis, putatively due to the modulation of microglial activation via inhibiting TLR4/NFκB/NLRP3 signaling pathway. Noteworthy, depletion of microglia eliminated the motor functional improvements after iTBS treatment. In the future, long-term effects of different rTMS regimens are necessary to further explore optimally response to neuronal damage and deleterious neuroinflammation.

## Supplementary Information

Below is the link to the electronic supplementary material.

**Additional file 1: Figure S1. (A)** The magnetic stimulator with a maximum magnetic stimulation intensity of 6 Tesla and a maximum change rates of magnetic induction intensity of 80 kT/s. **(B)** The regional cerebral blood flow of middle cerebral artery was monitored by laser speckle flowmetry. Representative images of double-labeling immunostaining of EdU/NeuN (EdU-green and NeuN-red) showed the neurogenesis in hippocampal dentate gyrus (DG) (Scale bar = 40 μm) **(C)** and subventricular zone (SVZ) (Scale bar = 60 μm) **(D)**, respectively. Quantitative analysis showed that iTBS significantly enhanced neurogenesis in DG and SVZ **(E)**. Values are expressed as the mean and 95% confidence interval (*n* = 4). Non-significant (ns), *P < 0.05, **P < 0.01 as determined by one-way ANOVA (Tukey's multiple comparison test). **(F)** Immunofluorescence staining for GFAP/TUNEL (GFAP-red and TUNEL-green) showed the activation of astrocytes over a time course from D1 to D7 after cerebral I/R injury (*n* = 4). Scale bar = 300 μm. **Figure S2. (A)** Immunofluorescence staining for NeuN/IL-1β colocalization (NeuN-red and IL-1β-green) among Sham, MCAO/r and iTBS groups. Scale bar = 40 μm. **(B)** Quantitative analysis of cell counts showed that iTBS significantly reduced the number of IL-1β positive cells in peri-infarcted area. **(C)** Colocalization analysis revealed that iTBS significantly reduced the expression of IL-1β on neurons in the peri-infarcted area compared with MCAO/r group. Values are expressed as the mean and 95% confidence interval (*n* = 4). *P < 0.05, **P < 0.01 as determined by one-way ANOVA (Tukey's multiple comparison test). 21-day PLX3397 treatment had also no significant impact on mean velocity **(D)** in open filed test, average speed **(E)**, stand **(F)**, swing **(G)** in CatWalk gait analysis. Values are expressed as the mean ± SEM of the mean (*n* = 6). Non-significant (ns), *P < 0.05, **P < 0.01 as determined by unpaired Student’s t test. **Table.** Details of the antibodies used in the experiment.

## Data Availability

All data analyzed and presented in this study are available from the corresponding author on reasonable request.

## Abbreviations

rTMS: Repetitive transcranial magnetic stimulation
iTBS: Intermittent theta-burst stimulation
MCAO/r: Middle cerebral artery occlusion/reperfusion
I/R: Ischemia/reperfusion
CNS: Central nervous system
cTBS: Continuous theta-burst stimulation
tDCS: Transcranial direct current stimulation
tFUS: Transcranial focused ultrasound stimulation
mNSS: Modified neurological severity score
IL: Interleukin
GSDMD: Gasdermin D
ASC: Apoptosis-associated speck-like protein containing CARD
NLRP1/3: NOD-like receptor protein 1/3
MAP2: Microtubule association protein 2
Iba-1: Ionized calcium binding adaptor molecule-1
GFAP: Glial fibrillary acidic protein
NeuN: Neuron-specific nucleoprotein
iNOS: Inducible nitric oxide synthase
Arg1: Arginase1
TLR4: Toll-like receptor 4
NFκB: Nuclear factor kappa-B
IFN-γ: Interferon γ
TNF-α: Tumor necrosis factor-α
MCP-1: Macrophage chemoattractant protein-1
DAPI: 4′,6′ Diamidino-2-phenylindole dihydrochloride hydrate
GO: Gene ontology
KEGG: Kyoto encyclopedia of genes and genomes
EdU: 5-Ethynyl-2′-deoxyuridine
TUNEL: Terminal deoxynucleotidyl transferase-mediated dUTP nick end labeling
TTC: 2,3,5-Triphenyltetrazolium chloride
