# Supplementary material for: Intermittent theta-burst stimulation improves motor function by inhibiting neuronal pyroptosis and regulating microglial polarization via TLR4/NFκB/NLRP3 signaling pathway in cerebral ischemic mice
Source: J Neuroinflammation. 2022 Jun 11;19:141. doi: 10.1186/s12974-022-02501-2 (PMC9188077; doi:10.1186/s12974-022-02501-2)
Supplement: Supplementary file 1 — Additional file 1: Figure S1. (A) The magnetic stimulator with a maximum magnetic stimulation intensity of 6 Tesla and a maximum change rates of magnetic induction intensity of 80 kT/s. (B) The regional cerebral blood flow of middle cerebral artery was monitored by laser speckle flowmetry. Representative images of double-labeling immunostaining of EdU/NeuN (EdU-green and NeuN-red) showed the neurogenesis in hippocampal dentate gyrus (DG) (Scale bar = 40 μm) (C) and subventricular zone (SVZ) (Scale bar = 60 μm) (D), respectively. Quantitative analysis showed that iTBS significantly enhanced neurogenesis in DG and SVZ (E). Values are expressed as the mean and 95% confidence interval (n = 4). Non-significant (ns), *P < 0.05, **P < 0.01 as determined by one-way ANOVA (Tukey's multiple comparison test). (F) Immunofluorescence staining for GFAP/TUNEL (GFAP-red and TUNEL-green) showed the activation of astrocytes over a time course from D1 to D7 after cerebral I/R injury (n = 4). Scale bar = 300 μm. Figure S2. (A) Immunofluorescence staining for NeuN/IL-1β colocalization (NeuN-red and IL-1β-green) among Sham, MCAO/r and iTBS groups. Scale bar = 40 μm. (B) Quantitative analysis of cell counts showed that iTBS significantly reduced the number of IL-1β positive cells in peri-infarcted area. (C) Colocalization analysis revealed that iTBS significantly reduced the expression of IL-1β on neurons in the peri-infarcted area compared with MCAO/r group. Values are expressed as the mean and 95% confidence interval (n = 4). *P < 0.05, **P < 0.01 as determined by one-way ANOVA (Tukey's multiple comparison test). 21-day PLX3397 treatment had also no significant impact on mean velocity (D) in open filed test, average speed (E), stand (F), swing (G) in CatWalk gait analysis. Values are expressed as the mean ± SEM of the mean (n = 6). Non-significant (ns), *P < 0.05, **P < 0.01 as determined by unpaired Student’s t test. Table. Details of the antibodies used in the experiment. [file 12974_2022_2501_MOESM1_ESM.docx]

**
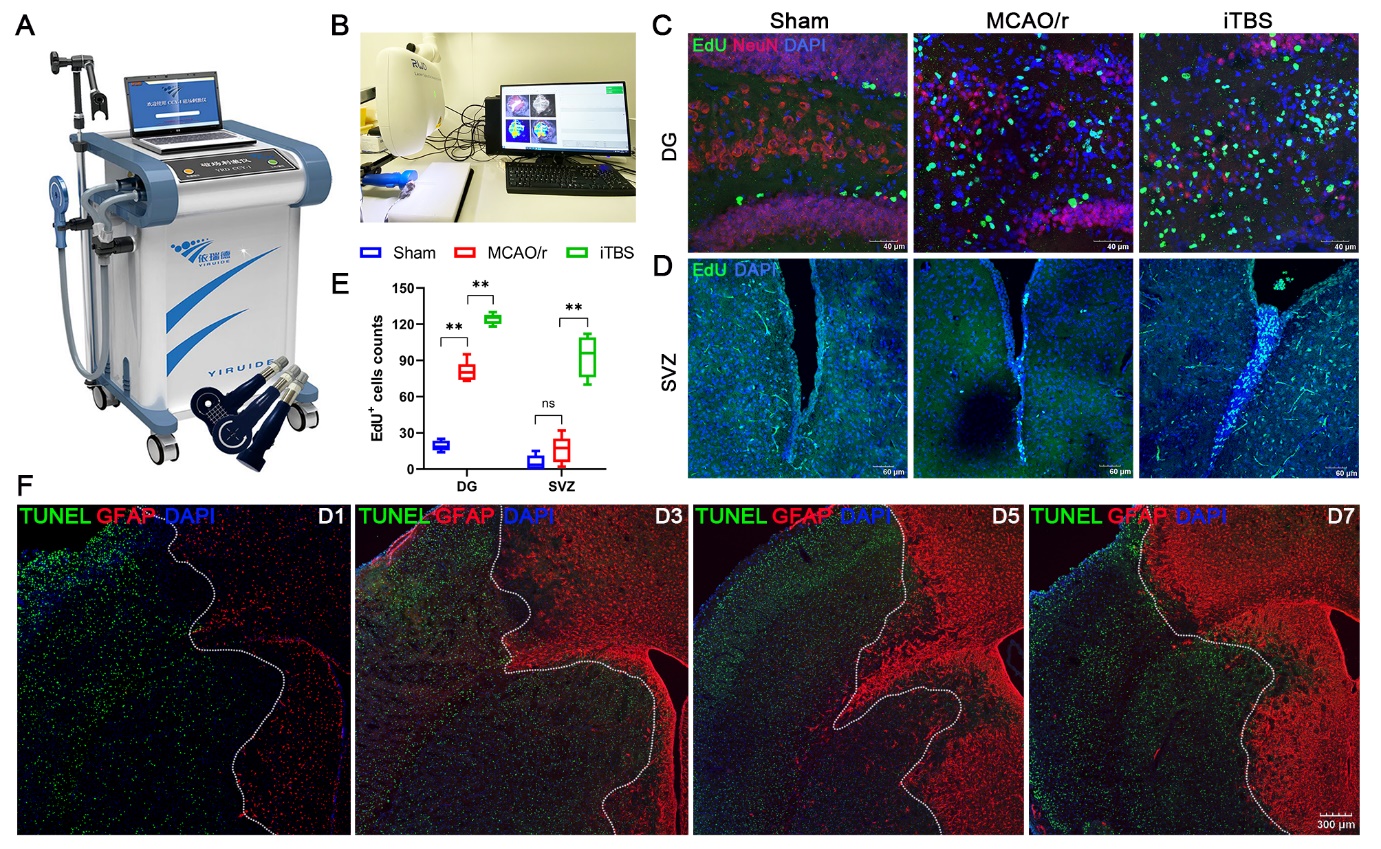
**

**Supplementary Fig. 1. (A)** The magnetic stimulator with a maximum magnetic stimulation intensity of 6 Tesla and a maximum change rates of magnetic induction intensity of 80 kT/s. **(B)** The regional cerebral blood flow of middle cerebral artery was monitored by laser speckle flowmetry. Representative images of double-labeling immunostaining of EdU/NeuN (EdU-green and NeuN-red) showed the neurogenesis in hippocampal dentate gyrus (DG) (Scale bar = 40 μm) **(C)** and subventricular zone (SVZ) (Scale bar = 60 μm) **(D)**, respectively. Quantitative analysis showed that iTBS significantly enhanced neurogenesis in DG and SVZ **(E)**. Values are expressed as the mean and 95% confidence interval (n = 4). Non-significant (ns), **P* < 0.05, ***P* < 0.01 as determined by one-way ANOVA (Tukey's multiple comparison test). **(F)** Immunofluorescence staining for GFAP/TUNEL (GFAP-red and TUNEL-green) showed the activation of astrocytes over a time course from D1 to D7 after cerebral I/R injury (n = 4). Scale bar = 300 μm.

**
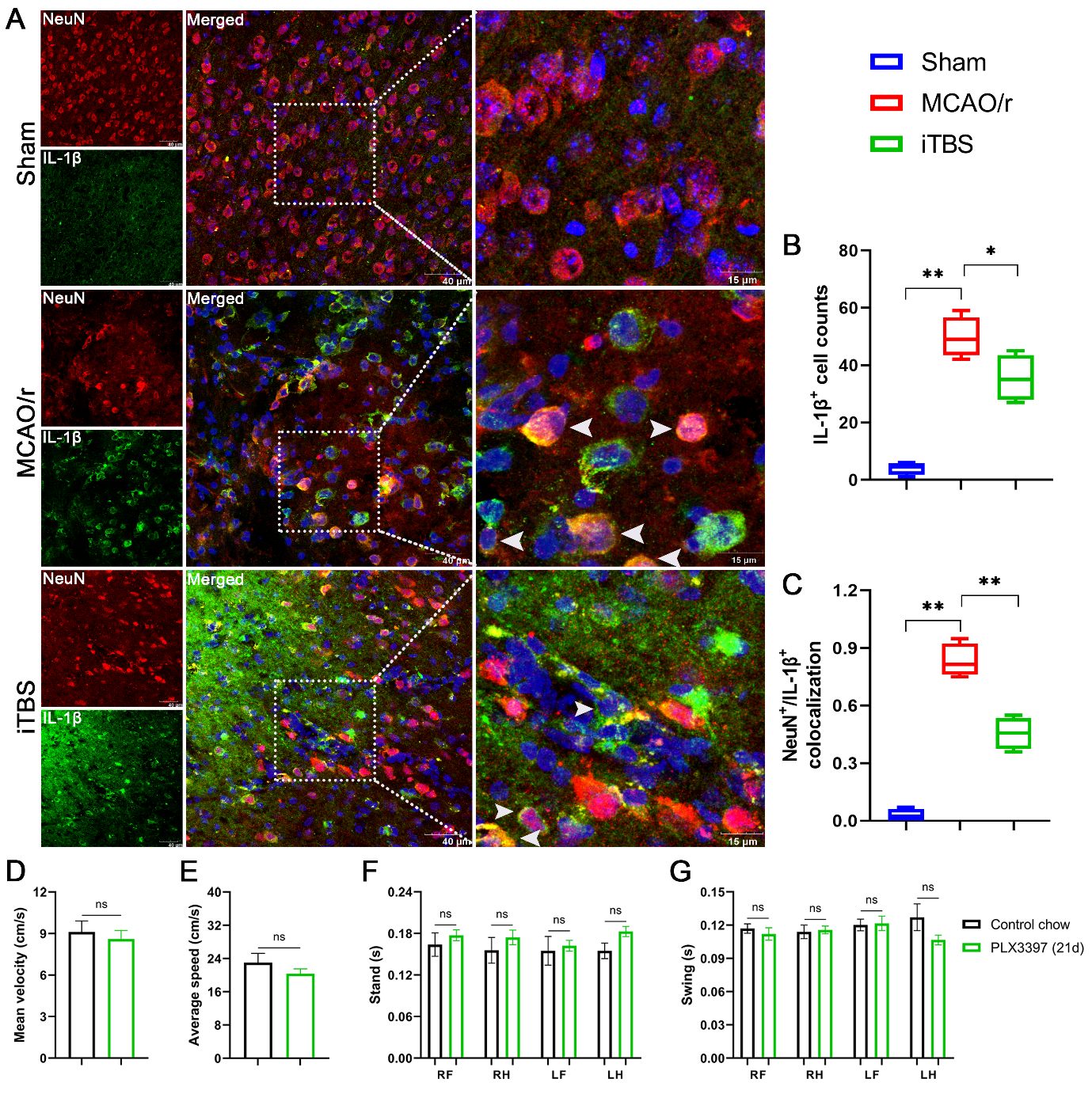
**

**Supplementary Fig. 2. (A)** Immunofluorescence staining for NeuN/IL-1β colocalization (NeuN-red and IL-1β-green) among Sham, MCAO/r and iTBS groups. Scale bar = 40 μm. **(B)** Quantitative analysis of cell counts showed that iTBS significantly reduced the number of IL-1β positive cells in peri-infarcted area. **(C)** Colocalization analysis revealed that iTBS significantly reduced the expression of IL-1β on neurons in the peri-infarcted area compared with MCAO/r group. Values are expressed as the mean and 95% confidence interval (n = 4). **P* < 0.05, ***P* < 0.01 as determined by one-way ANOVA (Tukey's multiple comparison test). 21-day PLX3397 treatment had also no significant impact on mean velocity **(D)** in open filed test, average speed **(E)**, stand **(F)**, swing **(G)** in CatWalk gait analysis. Values are expressed as the mean ± SEM of the mean (n = 6). Non-significant (ns), **P* < 0.05, ***P* < 0.01 as determined by unpaired Student’s t test.

**Supplementary Table. Details of the antibodies used in the experiment.**

| Antibody | Brand | Catalog number | Host | Dilution |
| --- | --- | --- | --- | --- |
| MAP2 | Proteintech | 17490-1-AP | Rabbit | 1:300 (IF) |
| GSDMD | Santa Cruz | sc-393581 | Mouse | 1:500 (WB)  1:300 (IF) |
| ASC | Santa Cruz | sc-514414 | Mouse | 1:500 (WB)  1:300 (IF) |
| NLRP1 | Novus Biologicals | NBP1-54899 | Rabbit | 1:1000 (WB)  1:300 (IF) |
| Caspase1 | Santa Cruz | sc-392736 | Mouse | 1:300 (IF) |
| pre.Caspase1 | Affinity | AF5418 | Rabbit | 1:1000 (WB) |
| cl.Caspase1 | Affinity | AF4005 | Rabbit | 1:1000 (WB) |
| pre.IL-1β | Affinity | AF5103 | Rabbit | 1:1000 (WB)  1:300 (IF) |
| cl.IL-1β | Affinity | AF4006 | Rabbit | 1:1000 (WB) |
| IL-18 | Affinity | DF6252 | Rabbit | 1:1000 (WB) |
| Iba1 (IF) | Wako | 011-27991 | Goat | 1:300 |
| Iba1 (WB) | Proteintech | 10904-1-AP | Rabbit | 1:1000 |
| CD86 | Proteintech | 13395-1-AP | Rabbit | 1:1000 (WB)  1:300 (IF) |
| CD206 | Abcam | ab64693 | Rabbit | 1:1000 (WB)  1:800 (IF) |
| iNOS | Santa Cruz | sc-7271 | Mouse | 1:500 (WB) |
| Arg1 | Santa Cruz | sc-271430 | Mouse | 1:500 (WB) |
| TLR4 | Santa Cruz | sc-293072 | Mouse | 1:500 (WB) |
| NLRP3 | AdipoGen | AG-20B-0014 | Mouse | 1:800 (WB) |
| NFκB | Santa Cruz | sc-8008 | Mouse | 1:500 (WB) |
| p-NFκB | Santa Cruz | sc-166748 | Mouse | 1:500 (WB) |
| GFAP | Proteintech | 16825-1-AP | Rabbit | 1:1000 (WB)  1:300 (IF) |
| NeuN | Proteintech | 26975-1-AP | Rabbit | 1:1000 (WB)  1:300 (IF) |
| β-actin | Bioss | bs-0061R | Rabbit | 1:4000 (WB) |
| Goat Anti-Rabbit IgG H&L antibody | Bioss | bs-0295G | Goat | 1:5000 (WB) |
| Goat Anti-Mouse IgG H&L antibody | Bioss | bs-0296G | Goat | 1:5000 (WB) |
| Goat anti-Mouse IgG (H+L), Alexa Fluor Plus 488 | Thermofisher | A32723TR | Goat | 1:800 (IF) |
| Donkey anti-Rabbit IgG (H+L), Alexa Fluor Plus 594 | Thermofisher | A32754 | Donkey | 1:800 (IF) |
| Donkey anti-Goat IgG (H+L), Alexa Fluor Plus 647 | Thermofisher | A21447 | Donkey | 1:800 (IF) |
